# Intrinsic Differences in Spatiotemporal Organization and Stromal Cell Interactions Between Isogenic Lung Cancer Cells of Epithelial and Mesenchymal Phenotypes Revealed by High-Dimensional Single-Cell Analysis of Heterotypic 3D Spheroid Models

**DOI:** 10.3389/fonc.2022.818437

**Published:** 2022-04-22

**Authors:** Maria L. Lotsberg, Gro V. Røsland, Austin J. Rayford, Sissel E. Dyrstad, Camilla T. Ekanger, Ning Lu, Kirstine Frantz, Linda E. B. Stuhr, Henrik J. Ditzel, Jean Paul Thiery, Lars A. Akslen, James B. Lorens, Agnete S. T. Engelsen

**Affiliations:** ^1^ Centre for Cancer Biomarkers (CCBIO), Department of Clinical Medicine, Faculty of Medicine, University of Bergen, Bergen, Norway; ^2^ Department of Biomedicine, Faculty of Medicine, University of Bergen, Bergen, Norway; ^3^ Department of Pathology, Haukeland University Hospital, Bergen, Norway; ^4^ BerGenBio, Bergen, Norway; ^5^ Institute of Molecular Medicine, University of Southern Denmark, Odense, Denmark; ^6^ Department of Oncology, Odense University Hospital, Odense, Denmark; ^7^ Guangzhou Laboratory, Guangzhou, China; ^8^ Gustave Roussy Cancer Campus, UMR 1186, Inserm, Université Paris-Saclay, Villejuif, France; ^9^ Department of Clinical Medicine, Section for Pathology, Faculty of Medicine, University of Bergen, Bergen, Norway

**Keywords:** non-small cell lung cancer (NSCLC), drug resistance, erlotinib-resistance, heterotypic 3D models, *in vitro* cell culture models, tumor microenvironment, imaging mass cytometry

## Abstract

The lack of inadequate preclinical models remains a limitation for cancer drug development and is a primary contributor to anti-cancer drug failures in clinical trials. Heterotypic multicellular spheroids are three-dimensional (3D) spherical structures generated by self-assembly from aggregates of two or more cell types. Compared to traditional monolayer cell culture models, the organization of cells into a 3D tissue-like structure favors relevant physiological conditions with chemical and physical gradients as well as cell-cell and cell-extracellular matrix (ECM) interactions that recapitulate many of the hallmarks of cancer *in situ*. Epidermal growth factor receptor (EGFR) mutations are prevalent in non-small cell lung cancer (NSCLC), yet various mechanisms of acquired resistance, including epithelial-to-mesenchymal transition (EMT), limit the clinical benefit of EGFR tyrosine kinase inhibitors (EGFRi). Improved preclinical models that incorporate the complexity induced by epithelial-to-mesenchymal plasticity (EMP) are urgently needed to advance new therapeutics for clinical NSCLC management. This study was designed to provide a thorough characterization of multicellular spheroids of isogenic cancer cells of various phenotypes and demonstrate *proof-of-principle* for the applicability of the presented spheroid model to evaluate the impact of cancer cell phenotype in drug screening experiments through high-dimensional and spatially resolved imaging mass cytometry (IMC) analyses. First, we developed and characterized 3D homotypic and heterotypic spheroid models comprising EGFRi-sensitive or EGFRi-resistant NSCLC cells. We observed that the degree of EMT correlated with the spheroid generation efficiency in monocultures. In-depth characterization of the multicellular heterotypic spheroids using immunohistochemistry and high-dimensional single-cell analyses by IMC revealed intrinsic differences between epithelial and mesenchymal-like cancer cells with respect to self-sorting, spatiotemporal organization, and stromal cell interactions when co-cultured with fibroblasts. While the carcinoma cells harboring an epithelial phenotype self-organized into a barrier sheet surrounding the fibroblasts, mesenchymal-like carcinoma cells localized to the central hypoxic and collagen-rich areas of the compact heterotypic spheroids. Further, deep-learning-based single-cell segmentation of IMC images and application of dimensionality reduction algorithms allowed a detailed visualization and multiparametric analysis of marker expression across the different cell subsets. We observed a high level of heterogeneity in the expression of EMT markers in both the carcinoma cell populations and the fibroblasts. Our study supports further application of these models in pre-clinical drug testing combined with complementary high-dimensional single-cell analyses, which in turn can advance our understanding of the impact of cancer-stroma interactions and epithelial phenotypic plasticity on innate and acquired therapy resistance in NSCLC.

## Introduction

Lung cancer is a leading cause of cancer-related morbidity and mortality worldwide (1). The dismal prognosis of advanced non-small cell lung cancer (NSCLC), despite recent improvements in targeted therapies including checkpoint inhibition, highlights the need to develop and apply more relevant lung cancer models. These models can allow physiologically relevant studies of drug responses, tumor-stroma interactions, and therapy resistance mechanisms. Mutations that activate epidermal growth factor receptor (EGFR) are prevalent in NSCLC (2, 3), and patients with tumors harboring EGFR driver mutations generally respond well to initial treatment using EGFR inhibitors (EGFRi). However, most, if not all, cancers eventually develop acquired drug resistance against EGFRi, limiting the long-term benefit of the treatment (2, 4). Various resistance mechanisms have been proposed; the most common of which include secondary EGFR mutations (such as the T790M point mutation), activation of bypass signaling pathways (including amplification of the MET (c-Met) receptor), alterations in downstream signaling, or phenotypic changes including transformation to the small cell lung cancer (SCLC) subtype or epithelial-to-mesenchymal transition (EMT) (2, 4).

In addition to mediating EGFRi resistance, EMT is also a common mechanism of resistance to multiple other cancer therapies, including chemotherapy, radiation, targeted therapies, as well as immunotherapy (5, 6). EMT has further been associated with poor prognosis and aggressive features in many cancers, making EMT an attractive therapeutic target (6). During EMT, highly polarized and well-organized epithelial cells lose their apicobasal polarity and tight cell-cell adhesions while adapting more mesenchymal-like features, including spindle-like morphology as well as increased migratory and invasive properties (6–8). At the molecular level, EMT can be recognized by downregulation of epithelial markers such as CDH1 (E-cadherin) and upregulation of mesenchymal markers such as CDH2 (N-cadherin) and VIM (vimentin). Multiple transcription factors (TFs) are involved in EMT, the most recognized of these being SNAI1 (Snail), SNAI2 (Slug), Twist family BHLH transcription factor 1 (TWIST), and Zinc finger E-box binding homeobox 1 and 2 (ZEB 1 and 2) (6). EMT may also be reversed by the mesenchymal-to-epithelial transition (MET). The remarkable ability to adapt to challenging microenvironmental conditions and transit among the continuum of phenotypic states along the EMT spectrum is referred to as epithelial-to-mesenchymal plasticity (EMP) (6, 8, 9). Notably, it is acknowledged that intermediate states across the EMT spectrum exhibiting EMP are the most relevant states to promote tumor progression (7–11), and epithelial plasticity has recently been acknowledged as a cancer hallmark (12, 13).

EMT is closely regulated by the tumor microenvironment (TME). Indeed, EMT was first demonstrated *in vitro* when Greenburg and Hay showed that cells from several different adult and embryonic epithelia changed their polarity and gained characteristics of migrating mesenchymal cells when exposed to collagen gels (14). EMT was first proposed to be a crucial mechanism for the progression of carcinoma in the early nineties (15–18). It is now well known that components of the TME, such as transforming growth factor-beta (TGFβ) and collagen type 1, are significant inducers of EMT. It is now acknowledged that TME affects multiple steps of cancer progression, including initiation, metastasis, and therapy resistance (19, 20). Under healthy homeostatic conditions, the tissue microenvironment is considered tumor-suppressive (21). However, the tissue microenvironment of the developing malignant tumors is severely altered compared to healthy tissues and may serve to support tumorigenesis (19, 22–25). The TME comprises various cell types, including fibroblasts, pericytes, endothelial cells, and immune cells (22, 23). Fibroblasts exhibiting different phenotypes are the most abundant cells of the TME. Cancer-associated fibroblasts (CAFs) can contribute to multiple steps of cancer progression, including tumor growth, extracellular matrix (ECM) remodeling, metastasis, and conditioning of the “metastatic niche” (19, 20). CAFs have been shown to enhance the invasiveness of cells *in vitro* and enhance the metastatic potential *in vivo* (26–28). Stromal fibroblasts have also been implicated in acquired resistance to EGFRi in lung cancer (29), while other studies have demonstrated that CAFs can induce EMT in nearby carcinoma cells (30–33). For example, conditioned medium from cultured CAFs induced EMT and increased the migration and invasion of A549 and SK-MES-1 lung cancer cells *in vitro* (26). More specifically, hepatocyte growth factor (HGF) secreted by fibroblasts, including the MRC-5 cell line, has been shown to modulate EMT and motility of human and murine epithelial cells (34). Further, A549 lung cancer cells injected subcutaneously into mice with or without human CAFs showed that CAFs enhanced tumor growth *in vivo* (26). Another study analyzing data from 1084 breast cancer patients in The Cancer Genome Atlas (TCGA) observed a correlation between EMT, stemness, and samples with a high stromal index (35).

Cells of clonal origin in two-dimensional (2D) culture do not reflect the heterogeneity and complexity of tumors *in vivo* (36). Still, 2D cultures are widely applied in pre-clinical drug screening experiments. Indeed, the simplistic nature of these cell-based models has also been considered one of the causes of failure in the translation of novel drugs and treatment regimens from the lab to the clinic (36–39). Thus, the application of more relevant *in vitro* and *in vivo* models is needed to improve pre-clinical to clinical translation. Improved *in vitro* models are complementary to the *in vivo* models. *In vitro* models may in some cases be a more applicable model for certain mechanistic studies, for example, when studying signaling pathways and cell type-specific events which are easier to achieve in a controlled *in vitro* setting relative to a more complex *in vivo* environment. In addition, advanced *in vitro* model systems can reduce the application of *in vivo* animal models and will be especially useful in eliminating further *in vivo* studies of compounds working in a concentration range that are expected to cause severe side effects.

Multiple strategies for studying cells *in vitro* in more relevant settings are being developed, and the use of multicellular spheroid models were pioneered in the 70ies (40–42). The term “spheroid” refers to the spherical geometry, and the term could in principle be applied for all types of sphere-like three-dimensional (3D) cultures. However, spheroids are commonly defined as 3D cultures generated by cells clustering or self-aggregating, often without the need of a scaffolding matrix (38, 43, 44). Spheroids can be developed from multiple sources such as cells harvested from human tissues or tumors, or by self-assembly of one or multiple different cell lines. The size and structure of the spheroids depend on the number of cells cultured and the cell types and their ability to establish cell-cell adhesions and potential to self-sort or compartmentalize depends on various factors, including the efficiency of binding between adhesion molecules expressed by the various cell types and the deposition extracellular matrix (38, 44–48). In addition to ‘spheroids’, ‘organoids’, ‘explant cultures’, and other 3D models are in use or in development, including the more complex “organ on a chip” models and 3D printing or 3D bioprinting approaches. For a comprehensive review of pros and cons for the different 3D cell culturing models, we refer the readers to a number of excellent review papers (36, 42, 44, 49–53).

In the present study, we have established and applied a 3D model where homotypic or heterotypic multicellular spheroids are formed by the self-aggregation of cells in ultra-low attachment plates (46). This spheroid model has the advantage of being relatively easy to work with and allow the study of a pre-defined composition and ratio of cancer and stromal cells. The model represents many aspects of the tissue or tumor of interest, including 3D geometry, chemical and physical gradients such as oxygen and nutrient availability and stiffness, cell-cell and cell-ECM interactions (36, 39, 51, 52). EGFRi resistance mechanisms and the impact of the cancer stroma interactions between fibroblasts and drug-sensitive or drug-resistant cancer cells remain largely unknown. Therefore, this study aimed to develop and characterize a 3D heterotypic co-culture system consisting of EGFRi-sensitive or EGFRi-resistant NSCLC cells together with fibroblasts that can be applied to study the impact of cancer-stroma interactions and EMP on acquired drug resistance in NSCLC.

## Materials and Methods

### Cell Culture

The human NSCLC cell line HCC827 (#CRL-2868, ATCC, Manassas, VA) was cultured in Roswell Park Memorial Institute (RPMI) 1640 medium (R8758, Sigma-Aldrich) containing 5% heat-inactivated Fetal Bovine Serum (FBS) (Gibco), 20 Units/mL Penicillin, 20 µg/mL Streptomycin (Penicillin-Streptomycin, #P-0781, Sigma-Aldrich) and 2 mM L-glutamine (#G-0781, Sigma-Aldrich). The HCC827 cell line has an activating EGFR mutation (E746–A750 deletion) that engenders sensitivity to erlotinib, and erlotinib-resistant (ER) clones of HCC827 cells were established *in vitro* by culture in increasing concentrations of erlotinib, as described previously (54, 55). The ER3 cells were kindly provided by Professor Trever G. Bivona at the Division of Hematology/Oncology, Helen Diller Comprehensive Cancer Center, University of California, San Fransisco, CA, USA (54). The ER10, ER20, and ER30 cells were established at the University of Southern Denmark (55). Erlotinib-resistant cells were maintained in culture with 1 µM erlotinib (#5083S, Cell Signaling Technology, Danvers, MA, USA). It has previously been shown by sequencing that the ER3 clone does not harbor the common EGFR T790M point mutation (54). For the ER10, ER20 and ER30 clones, it has been verified that the EGFRdel19 mutation is preserved in all resistant clones, while no other known EGFR or KRAS driver mutations were detected (including EGFR T790M) (55). Next-generation sequencing revealed a *TP53* V218del mutation, and *EGFR* and *CDK4* amplification in all clones including the parental cell line (55). In addition, *HER2* amplification was detected in ER10 (6.2 copies) and *MET* amplification in ER30 (8.3 copies) (55).

The human NSCLC cell line H1975 and rociletinib (CO-1686)-resistant (COR) clones of this cell line; COR1-1 and COR10-1 were provided by Clovis oncology (Clovis oncology, Boulder, Colorado, US). H1975 cells were cultured in the same way as HCC827 cells, and the COR clones were supplemented with 1 µM rociletinib (CO-1686, Clovis oncology).

The human fibroblast cell line SV80 (SV40 transformed) (CLS Cell Lines Service GmbH, Eppelheim, Germany) was cultured in Dulbecco′s Modified Eagle′s Medium/Nutrient Mixture F-12 (DMEM/F12) (D8062, Sigma) supplemented with 5% FBS (Gibco), 20 Units/mL Penicillin, 20 µg/mL Streptomycin (Penicillin-Streptomycin, #P-0781, Sigma-Aldrich) and 2 mM L-glutamine (#G-0781, Sigma-Aldrich).

The human lung fibroblast cell line MRC-5 (ATCC, CCL-171) was cultured in Dulbecco′s Modified Eagle′s Medium (DMEM) (D5671, Sigma) containing 5% FBS (Gibco), 20 Units/mL Penicillin, 20 µg/mL Streptomycin (Penicillin-Streptomycin, #P-0781, Sigma-Aldrich) and 2 mM L-glutamine (#G-0781, Sigma-Aldrich).

Cell lines were routinely tested for Mycoplasma contamination using the MycoAlert Mycoplasma Detection Kit (LT07–218, Lonza, Basel, Switzerland). All cell lines used in these studies were authenticated by short tandem repeat (STR) profiling using the LGC service Promega’s PowerPlex 18D System. The American Association for Cancer Research (AACR) STR database (135-XV-5, ATCC) was applied as a reference for authentication of STR profiles. All cells were maintained in a humidified incubator at 37°C, 5% CO_2_ and 5% O_2_. Cell culture conditions are summarized in **Table 1**.

**Table 1:** Cell culturing conditions.

Name	Short name	Cell type and origin	Supplier	Cell culture medium	Cell culture drug supplement
**HCC827 parental**	HCC827	Human NSCLC epithelial cell line	#CRL-2868, ATCC (HCC827)	RPMI1640 (R8758, Sigma)*	NA
**HCC827 ER3,**	ER3,	Erlotinib-resistant clones of HCC827	#CRL-2868, ATCC (HCC827) ^1,2^	RPMI1640 (R8758, Sigma)*	1µM erlotinib
**HCC827 ER10,**	ER10,				
**HCC827 ER20,**	ER20,				
**HCC827 ER30**	ER30				
**H1975 parental**	H1975	Human NSCLC (adenocarcinoma) epithelial cell line	#CRL-5908, ATCC (H1975)	RPMI1640 (R8758, Sigma)*	NA
**H1975 COR1-1, H1975 COR10-1**	COR1-1,	Rociletinib-resistant clones of H1975	#CRL-5908, ATCC (H1975) ^3^	RPMI1640 (R8758, Sigma)*	1µM rociletinib
	COR10-1				
**SV80**	SV80	Human lung fibroblast (SV40-transformed cell line)	CLS Cell Lines Service GmbH	DMEM/F12 (D8062, Sigma)*	NA
**MRC-5**	MRC-5	Human lung fibroblasts	#CCL-171, ATCC	DMEM (D5671, Sigma)*	NA

^1^ER3 clone provided by Professor Trever G. Bivona at the Division of Hematology/Oncology, Helen Diller Comprehensive Cancer Center, University of California, San Fransisco, CA, USA (Zhang et al., 2012).

^2^ER10, ER20, and ER30 clones were developed at the Institute of Molecular Medicine, University of Southern Denmark, Odense, Denmark.

^3^Rociletinib-resistant clones provided by Clovis oncology.

*All cell culture media were also supplemented with 5% heat-inactivated FBS (Gibco), 20 Units/ml Penicillin, 20 ug/ml Streptomycin (Penicillin-Streptomycin, #P-0781, Sigma) and 2 mM L-glutamine (#G-0781, Sigma).

NA, not applicable.

### 3D Spheroid Formation by Mono- and Co-Culturing of NSCLC Cells and Fibroblasts

3D cultures were established by seeding cancer cells alone (9 000 cells per well) or cancer cells and fibroblasts (SV80 or MRC-5) in a 1:2 ratio (3 000 cancer cells and 6 000 fibroblasts) in a final volume of 100 µL cell culture medium per well of 96-well round-bottom ultra-low attachment plates (#7007, Corning Inc., Corning, NY, US). To encourage efficient generation of multicellular spheroids, the plates were centrifuged at 1019 g for 20 min, and thereafter placed in a cell culture incubator at 37°C, 5% CO_2_, 5% O_2_. The next day, an additional 100 µL fresh cell medium was added per well. Monocultures were maintained in 200 µL RPMI1640 medium, while co-culture spheroids were cultured in equal volumes of DMEM and RPMI1640 supplemented with FBS, penicillin, streptomycin, and L-glutamine, as described. Culture media were changed every second to every third day by carefully removing 100 µL and adding 100 µL fresh medium. Phase object confluence was used as a surrogate parameter representing the quantification of spheroid formation. Generation of compact spheroid structures was quantified using the IncuCyte ZOOM microscope and the built-in software. Masking was performed using the following settings: segmentation adjustment: 1, Hole fill: 30 000 µm^2^, Filtered minimum area: 5 000 µm^2^.

### Gene Expression Analysis

To prepare samples for gene expression analysis, cells were collected by scraping in ice-cold phosphate-buffered saline (PBS), pelleted by centrifugation and immediately frozen at –80°C. Ribonucleic Acid (RNA) was harvested using the RNeasy MINI KIT (74104, QIAGEN, Venlo, Netherlands) according to the manufacturer’s protocol. Deoxyribonucleic Acid (DNA) was removed using the RNase-Free DNase set (79254, QIAGEN, Venlo, Netherlands). Complementary DNA (cDNA) was made by High-Capacity cDNA Reverse Transcription Kit (4368813, Thermo Fisher Scientific, Waltham, MA) according to the manufacturer’s instructions. Real-time quantitative Polymerase Chain Reaction (RT-qPCR) was performed with three technical replicates of 1 µL and 5 µL of total volume as described in Dyrstad et al. (56) with the following probes from Applied Biosystems (Thermo Fisher Scientific, Waltham, MA); *AXL* (hs01064444), *CDH1* (hs01023894), *CDH2* (hs00983056), *VIM* (hs00185584), *GAS6* (hs01090305) and Eukaryotic *18S* rRNA Endogenous Control (4310893E). Fold change gene expression was calculated by the 2^–ΔΔCt^ method normalizing against the gene expression in HCC827 parental cells.

### Western Blotting

To prepare lysates for western blotting, cells were washed with ice-cold PBS, collected by scraping in PBS on ice, pelleted by centrifugation, and lysed in Radioimmunoprecipitation assay (RIPA) buffer (sc-24948A, Santa Cruz Biotechnology, Dallas, TX). Protein concentration was measured using Pierce BCA Assay Kit (#23225/23227, Thermo Fisher Scientific, Waltham, MA). Lysates were dissolved in water with BIO-RADxT sample buffer (#1610791, Bio-Rad, Hercules, CA), incubated for 5 min at 90°C and collected by centrifugation. 10 µg protein were loaded per well on 4%-20% Mini-PROTEAN TGX Stain-Free Gels (#4561096, Bio-Rad). The proteins were separated by electrophoresis at 90 V for 10 min, followed by 130 V for 1 h. To allow total protein quantification, the stain-free gels were activated by 2.5 min exposure to ultraviolet (UV) light (ChemiDoc XRS+, Bio-Rad). Semi-dry blotting to a Trans-blot turbo mini-size low-fluorescence polyvinylidene difluoride (LF PVDF) membrane (#1704156, Bio-Rad) was performed was performed using the Bio-Rad Trans-Blot system (2.5 A, 25 V, 7 min). Tris-Glycine SDS (TGS) buffer (#1610772, Bio-Rad) was used for the transfer. Images of the total protein amount of the membrane were captured directly after the transfer, and these images were later used for normalization against total protein for further quantifications of western blots as described for stain-free gels by the manufacturer (ChemiDoc XRS+, Bio-Rad) and by Gürtler et al. (57). Membranes were blocked with 5% Bovine Serum Albumin (BSA) (A2058, Sigma-Aldrich) or 5% non-fat dry milk in tris-buffered saline (TBS) with 0.1% Tween-20 (TBS-T) and incubated with primary antibody overnight at 4°C. Primary antibodies used: anti-CDH1 (1:1000, 14472S, Cell Signaling Technology); anti-CDH2 (1:500, ab18203, Abcam); anti-VIM (1:5000, ab92547, Abcam). Membranes were washed 3x for 5 min with TBS-T and incubated for 1 h at room temperature with 1:10,000 of goat anti-mouse Horseradish Peroxidase (HRP) (#170-6516, Bio-Rad) or goat-anti-rabbit HRP (#170-6515, Bio-Rad) secondary antibodies. Chemiluminescent substrate was added (Super Signal West Femto Maximum Sensitivity Substrate, #34095, Thermo Fisher Scientific), and chemiluminescence was measured by Molecular Imager ChemiDoc XRS+ (Bio-Rad). The ImageLab v5.0 (Bio-Rad) software was used for analysis and quantification of the results.

### Immunocytochemistry and Confocal Imaging

For immunocytochemistry (ICC), 20,000 cells/well were seeded in 24-well plates containing crystal clear German glass coverslips with Poly-D-lysine coating (#GG-12-PDL, Neuvito Corporation, Vancouver, WA). The cells were allowed to attach to the coverslips overnight. Cells were washed in PBS and fixed with 3.7% formalin for 15 min at room temperature. The cells were then washed with PBS with 0.1% Tween-20 (PBS-T), permeabilized with 0.3% Triton X-100 for 20 min, and blocked with 5% goat serum (G9023, Sigma-Aldrich) for one hour at room temperature. Incubation with primary antibody against CDH1 (#14472S, Cell Signaling Technology, 1:100) and VIM (ab92547, Abcam, 1:100) or TUBA1A (ab7291, Abcam, 1:1000) overnight at 4°C was followed by washes and one hour incubation with secondary antibodies (goat anti-mouse AF488, 1:200, A11029, Thermo Fisher Scientific and goat anti-rabbit AF546, 1:200, A11035, Invitrogen, Carlsbad, CA) at room temperature. Cells were washed and mounted with ProLong™ Diamond Antifade Mountant with 4′,6-diamidino-2-phenylindole (DAPI) (#P36962, Molecular Probes, Eugene, OR). Images were obtained on a Leica TCS SP8 confocal microscope using 100x objective (HC PL Apo STED white, oil, NA = 1.4, WD = 0.13 mm).

### Lentiviral Expression Plasmids and Fluorescence-Activated Cell Sorting

Lentiviral expression plasmids encoding Enhanced Green Fluorescent Protein (EGFP) or Discosoma Red Fluorescent Protein (dsRed) were produced as previously described (58). Triple transfection of HEK293 packaging cells was performed with the expression plasmid, accompanied by the pMD2.G packaging plasmid and pVSV-G envelope plasmid (Tronolab) as described previously (59). Cell populations were sorted twice by fluorescence-activated cell sorting (FACS) (Sony SH800) to obtain cell populations containing high percentages of stably transduced cells with medium-high transgene expression. Stably transduced and FACS sorted cells were subsequently used for live-cell imaging.

### Characterization of Heterotypic Co-Culture Spheroids by Confocal Microscopy and Reconstruction in IMARIS

Heterotypic co-culture spheroids made of ER3-GFP (750 cells) and SV80-dsRed (1 500 cells) were harvested ten days post seeding, fixed, stained with Hoechst, and imaged by confocal microscopy. Briefly, the spheroids were collected by centrifugation and resuspended in 2 mL 3.7% formalin in PBS. The spheroids were incubated with the fixative for 10 min at room temperature. The spheroids were centrifuged and resuspended in 1 mL 1 µg/mL Hoechst solution in PBS. The spheroids were stained in the Hoechst solution at 4°C until microscopy was performed at a Zeiss confocal microscope eight days later. IMARIS was used to reconstruct the 3D z-stack images (18 images covering a depth of 71.77 µm).

### Paraffin Embedding of Spheroids

Heterotypic 3D cultures were harvested after seven days of culture and captured in either a clot made of fibrin and thrombin or in agarose gel before embedding in paraffin according to standard procedures. Briefly, the spheroids were fixed in 3.7% formalin overnight at room temperature. Spheroids were then collected by centrifugation and briefly counterstained with methyl green for 2 min. Following a wash to remove excess stain, the spheroids were captured in a blood clot or agarose gel as indicated in the figure legends. For clot capture, 20 µL of human blood plasma was added to the spheroids and mixed well to ensure good coating necessary for a successful cast when 10 µL of 100 U/mL thrombin was added to the plasma coated spheroids to make a coagulate. For embedding in agarose gel, spheroids were resuspended in 100 μL pre-warmed 1.5% agarose (Sigma, A9045) in TBS (Bio-Rad, #1706435), and incubated at 4°C for 30 min for the agarose gel to solidify. In both cases, the gel or clot containing spheroids were transferred to a cell safe biopsy capsule (CellPath, EBE-0201-02A, UK and Simport, M498-2, Canada) and stored in 70% ethanol until paraffin embedding according to standard protocols performed at the molecular imaging center (MIC) core facility at the Department of Biomedicine, University of Bergen. The paraffin-embedded spheroids were further sectioned by a microtome into 5 μm sections and collected at SuperFrost+ slides (10149870, Thermo Scientific). Spheroid sections were stained with Hematoxylin and Eosin (H&E) following standard procedures or processed for immunohistochemistry (IHC) or imaging mass cytometry (IMC), as described below. Images of the H&E stained sections were used for spheroid diameter quantification using the built-in measuring tool in Fiji (60, 61).

### Immunohistochemistry of Paraffin Embedded Tissue-Sections With Fluorescent Detection (IHC-P/IF)

Formalin-fixed paraffin-embedded (FFPE) slides were deparaffinized in xylene (2x 10 min) and hydrated in an ethanol (EtOH)-series of decreasing concentration. Briefly, 2x 5 min in AbsEtOH, 2x 5 min in 96% EtOH, 1x 5 min in 70% EtOH, 1x 5 min in 50% EtOH, and finally rehydrated in water (2x 5 min in Milli-Q water). Heat-induced antigen retrieval was conducted in DAKO Target Retrieval Solution (Dako, s1699) for 30 min at 95°C using a decloaking chamber (Decloaking Chamber NxGen, Biocare Medical). When the decloaking chamber cooled down and reached a temperature of 80°C, the slides in retrieval buffer solution were taken out and placed at room temperature for 20 min to cool down. Slides were then washed for 10 min in Milli-Q water and 10 min with PBS-T with gentle agitation. The remaining water was wiped off, and the tissue was encircled with a hydrophobic pen (DAKO, S2002, Glostrup, Denmark). Blocking buffer (5% BSA in PBS-T) was added immediately to avoid tissue from drying out, and slides were incubated in blocking buffer for 1 hour at room temperature in a humidity chamber. Subsequently, the slides were stained with primary antibody (**Table 2**) overnight at 4°C. The next day, slides were washed 3x5 min with PBS-T and incubated with fluorescence tagged secondary antibodies for 1 h at room temperature. The secondary antibodies were diluted 1:200 in 0.5% BSA in PBS-T. Slides were washed 3x in PBS and mounted with Prolong DAPI Diamond (P36962, Thermo Fisher Scientific). Samples stained with fluorescent secondary antibodies were imaged with a Zeiss Collibri7 microscope.

**Table 2:** Antibodies for western blot (WB), Immunocytochemistry with immunofluorescent detection (ICC/IF), and Immunohistochemistry of paraffin sections with immunofluorescent detection (IHC-P/IF).

Antibody target	Clone	Product number	Supplier*	Dilution	Application*
**CDH1 (E-cadherin)**	4A2	14472S	CST	1:1 000	WB
				1:100	ICC/IF
**CDH2 (N-cadherin)**	Polyclonal	ab18203	Abcam	1:500	WB
**VIM (Vimentin)**	EPR3776	ab92547	Abcam	1:5 000	WB
				1:100	ICC/IF
**TUBA1A (Alpha-tubulin)**	DM1A	ab7291	Abcam	1:1 000	ICC/IF
**EGFRdel (E746-A750del specific)**	D6B6	2085	CST	1:100	IHC-P/IF

*CST, Cell Signaling Technology; WB, Western Blot; ICC, Immunocytochemistry; IHC-P, Immunohistochemistry-Paraffin; IF, Immunofluoresence.

### Imaging Mass Cytometry (IMC)

Deparaffinization, antigen retrieval, and blocking were performed as described for (IHC-P/IF) above. Primary antibodies were either purchased in ready to use format from Fluidigm or conjugated with metal isotopes *in-house* using the Maxpar^®^ X8 Multimetal Labeling Kit (201300, Fluidigm). Antibodies were centrifuged for 2 min at 1000 g at 4°C to remove any potential precipitates of aggregated antibodies before all antibodies were added into a cocktail and diluted in 0.5% BSA in PBS as specified in **Table 3**. After blocking, the antibody cocktail was added to the tissue sections and incubated overnight at 4°C. The following day, the slides were washed for 2x8 min in PBS-T followed by 2x8 min washes in PBS. All washing steps were performed with gentle agitation. Subsequently, the tissue was stained with 250 μM Intercalator-Irridium (Ir, Fluidigm, 201192B) in PBS for 30 min at room temperature. The slides were washed for 5 min in PBS followed by 5 min Milli-Q water under gentle agitation. Slides were dried at room temperature and stored in a dust-free and dry container with desiccants until laser ablation by the Hyperion Imaging System (Fluidigm) coupled to a Helios time-of-flight mass spectrometer (Fluidigm). Five regions of interest (ROIs), each containing a single spheroid, were ablated for each sample. MCD-files created by the CyTOF^®^ Software v7.0 was processed in MCD viewer (Fluidigm) to create pseudo-colored images for marker expression visualization.

**Table 3:** List of IMC antibodies.

Metal tag	Antibody target	Clone	Product number	Supplier	Dilution*
141 Pr	ACTA2 (αSMA)	1A4	201505	Fluidigm	1:50
142 Nd	EGFR	D38B1	3142013D	Fluidigm	1:50
143 Nd	VIM (vimentin)	D21H3	201505	Fluidigm	1:400
144 Nd	phospho-Tyr	P-Tyr-100	3144024D	Fluidigm	1:50
145 Nd	Laminin	polyclonal	PA-16730	ThermoFisher	1:500
148 Nd	Cytokeratin^§§^	AE1/AE3	3148022D	Fluidigm	1:200
150 Nd	MUC1^§§^ (Mucin1/CD227)	SM3	3150032D	Fluidigm	1:50
151 Eu	TP63 (p63)	polyclonal	ab53039	Abcam	1:50
154 Sm	HIF1A (HIF-1 α)	EP1215Y	ab210073	Abcam	1:50
155 Gd	GFP	4B10B2	MA5-15349	ThermoFisher	1:100
158 Gd	CDH1 (E-cadherin)	24E10	201505	Fluidigm	1:100
159 Tb	RFP	Polyclonal	600-401-379	Rockland	1:50
161 Dy	MKI67 (Ki67)	B56	3161007B	Fluidigm	1:100
165 Ho	beta-catenin^§§^	D13A1	3165032D	Fluidigm	1:150
167 Er	MET^§§^	D1C2	3167020D	Fluidigm	1:50
169 Tm	Collagen 1	Polyclonal	201505	Fluidigm	1:400
171 Yb	Histone H3^§^	D1H2	201505	Fluidigm	1:4000
173 Yb	PDGFRB^§§^	28E1	3169	Cell Signaling Technology	1:50
175 Lu	pan-actin	D18C11	3175032D	Fluidigm	1:50
176 Lu	phospho-Histone H3^§§^	HTA28 [Ser28]	3176024D	Fluidigm	1:100
191 Ir	Intercalator-Ir	DNA	201192B	Fluidigm	250 μM
193 Ir	Intercalator-Ir	DNA	201192B	Fluidigm	250 μM

^§^Antibodies only included in the first IMC run with untreated samples.

^§§^Antibodies only included in the second IMC run with the erlotinib and DMSO treated samples.

*Stock concentration for in-house conjugated antibodies is 500 µg/ml.

### Single Cell Segmentation and High-Dimensional Analysis of IMC Data

Pre-processing and single-cell segmentation of IMC images were performed using the Steinbock pre-processing pipeline; https://bodenmillergroup.github.io/steinbock/v0.9.1/ (62). Each heavy metal channel comprises a staining intensity image of a single protein marker or DNA intercalator. All image channels were first subjected to a 99.5% upper threshold to remove high outlier pixels. Single-cell segmentation masks were generated using Mesmer and the pre-trained MultiplexSegmentation dataset (63), using Histone H3 or Ir191/193 to identify nuclei and GFP and RFP to identify cytoplasm. For the treated spheroid dataset, an additional distance-to-border image channel was generated as described previously (64). Briefly, a classifier was trained within the Ilastik software (65) to recognize pixels as either spheroid or background and the resulting probability images were exported into the CellProfiler software to make binary masks of the spheroid areas. These masks were then used to generate a distance-transformed image using the TransformBinary module (ImcPluginsCP, https://github.com/BodenmillerGroup/ImcPluginsCP), where the value of each pixel within the spheroid area represents the minimum distance to a non-spheroid (background) pixel. Mean pixel intensity data was extracted for each image channel for each cell in the segmentation masks. CSV files containing the corresponding single-cell data were exported for further downstream analysis using the Cytobank software. Single-cell data were then gated based upon GFP and RFP expression and marker expression was visualized in heatmaps generated in Cytobank or exported to GraphPad Prism for visualization of single markers in histograms, and the distance to border parameter was visualized in violin plots. The t-Distributed Stochastic Neighbor Embedding (tSNE) algorithm was applied on the ungated population using the Cytobank software (tSNE-CUDA advanced analysis). The following settings were used for the untreated samples: all events included (no downsampling, total events: 14 271), 750 iterations, perplexity = 30, learning rate automatic (1189), early exaggeration 12, random seed, scales of each tSNE channel normalized to have a mean of 0 with a standard deviation of 1, channels used in tSNE: EGFR, CDH1 (E-cadherin), and VIM (vimentin). The following settings were applied for the treated samples: equal downsampling (2,147 × 4 files = 8,588 total events), 750 iterations, perplexity = 30, learning rate automatic (715), early exaggeration 12, random seed, scales of each tSNE channel normalized to have a mean of 0 with a standard deviation of 1, channels used in tSNE: EGFR, CDH1 (E-cadherin), VIM (vimentin), MET (c-Met), pan-cytokeratin, GFP and RFP.

### Statistics

Data are presented as mean values +/- standard deviations (SD) or as fold changes from a representative experiment if not otherwise indicated in the figure legends. One-way Analysis of variance (ANOVA) followed by Tukey’s multiple comparisons test or non-parametric Mann-Whitney U-test was performed as described in the figure legends. Statistical analyses were performed using the GraphPad Prism software version 9.3.0 if otherwise is not stated in the figure legends. For the IMC data, statistical significance in absolute cell counts and channel expression values were calculated in cytobank using the Mann-Whitney U-test. The following symbols are given to report statistical significance: ns = *P* > 0.05, *P ≤ 0.05, **P ≤ 0.01, ***P ≤ 0.001, ****P ≤ 0.0001.

## Results

### Cell Models of Acquired Drug Resistance Against EGFR Targeted Therapies

**Figure 1:**
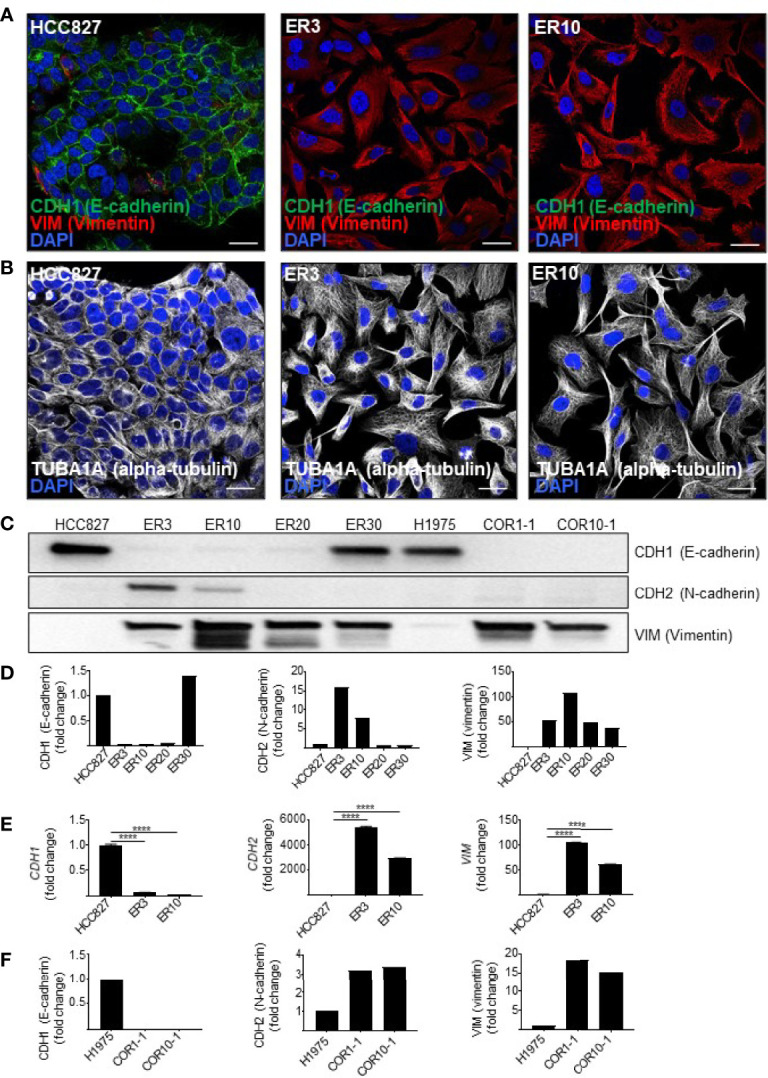
Resistance to first- and second-generation EGFR inhibitors is associated with features of EMT. **(A)** Immunocytochemistry of HCC827 parental cells and the erlotinib-resistant clones ER3 and ER10 for the epithelial marker CDH1 (E-cadherin) and the mesenchymal marker VIM (vimentin) to examine markers of epithelial plasticity upon acquired drug resistance. Counterstain by DAPI. Scalebar = 30 μm. **(B)** TUBA1A (alpha-tubulin) immunocytochemistry of the cells described in **(A)** were applied to reveal the phenotypic shift in cell morphology. Counterstain by DAPI. Scalebar = 30 μm. **(C)** Western blots were prepared with lysates from the HCC827 parental cells and the erlotinib-resistant clones ER3, ER10, ER20, and ER30 H1975 parental cells and the clones COR1-1 and COR10-1 resistant to the second-generation EGFR inhibitor rociletinib. Immunodetection of epithelial marker CDH1 (E-cadherin) (135 kDa), mesenchymal markers CDH2 (N-cadherin) (135 kDa), VIM (vimentin) (54 kDa). Western blot analysis was repeated n = 3 times, and a representative experiment is presented in the figure. **(D)** Quantification of the western blot presented in **(C)** normalized against total protein presented in **Supplementary Figure 1A** (VIM and CDH1) and B (CDH2). Fold change values for the resistant clones ER3 and ER10 relative to their parental cell line HCC827 **(E)** Expression of transcripts encoding *CDH1, CDH2, VIM*, assessed by RT-qPCR on cDNA prepared from HCC827 parental, ER3, and ER10 cells. RT-qPCR analyses were repeated n = 3 times, and representative results from one experiment with n = 3 technical replicates are presented in the figure as mean fold change +/- SD calculated by the 2^–ΔΔCt^ method. Two-way ANOVA followed by Tukey’s multiple comparison test comparing ER3 and ER10 against the parental cell line showed that for all genes, the gene expression of all genes in both ER3 and ER10 were significantly different from the parental cells (*P* < 0.0001). **(F)** Quantification of the western blot presented in **(C)** normalized against total protein presented in **Supplementary Figure 1A** (VIM and CDH1) and B (CDH2). Fold change values for the resistant clones COR1-1 and COR10-1 relative to their parental cell line H1975. ****P ≤ 0.0001.

To produce isogenic cell lines displaying a range of epithelial-mesenchymal phenotypes, several models were established. The human NSCLC cell line HCC827 harbors an activating EGFR mutation (E746–A750 deletion). This activating EGFR mutation renders the cells sensitive to EGFRi, including erlotinib. To generate a model of acquired resistance, HCC827 cells were exposed to erlotinib *in vitro* to generate erlotinib-resistant (ER) clones, as previously described (54, 55). In 2D culture, parental HCC827 cells display an epithelial phenotype characterized by the expression of CDH1 (E-cadherin). In contrast, the erlotinib-resistant clones ER3 and ER10 are characterized by upregulation of VIM (vimentin) and downregulation of CDH1 (E-cadherin) (**Figure 1A**). The cadherin-switch and upregulation of the mesenchymal marker VIM (vimentin) are characteristic features of cells that have undergone EMT and gained a more mesenchymal phenotype. Compared to the epithelial cobblestone-like morphology of parental HCC827 cells, the ER3 and ER10 cells display a mesenchymal-like spindle-shaped morphology as visualized by staining the cytoskeletal component TUBA1A (alpha-tubulin) (**Figure 1B**). The mesenchymal phenotype of ER3 and ER10 cells was confirmed by western blotting (**Figure 1C**, quantified in **Figure 1D**, corresponding total protein images used for normalization in **Figures S1A, B**). Transcriptional alterations in the genes encoding *CDH1*, *CDH2*, and *VIM* were also analyzed, and the pattern of downregulated *CDH1* and upregulated *CDH2* and *VIM* in ER3 and ER10 cells compared to parental HCC827 cells were confirmed at the transcriptional level by RT-qPCR (**Figure 1E**). In addition, the receptor tyrosine kinase *AXL* and its ligand *GAS6* were found to be transcriptionally upregulated in ER3 and ER10 cells compared to the parental cell line HCC827 (**Figure S1C**). The shift towards a mesenchymal phenotype upon erlotinib resistance, as observed in ER3 and ER10 cells, is consistent with previous findings from our laboratory and others (54, 66). Epithelial and mesenchymal markers were further assessed by western blotting for two additional erlotinib-resistant clones derived from the HCC827 cell line, namely ER20 and ER30 (**Figure 1C**, quantified in **Figure 1D**, corresponding total protein images used for normalization in **Figures S1A, B**). A prominent increase in VIM (vimentin) expression was observed in ER20 and ER30. However, the ER20 and ER30 clones did not display an increase in CDH2 (N-cadherin) expression as observed in ER3 and ER10. Furthermore, ER30 did not show downregulation of the epithelial marker CDH1 (E-cadherin), as observed in ER3, ER10 and ER20. It has been described previously by Terp and colleagues that ER30 carries a *MET* (c-Met) amplification which is a known resistance mechanism against EGFRi, indicating a genetic driver of resistance in the ER30 clone (55).

As EMT has also been established as a mechanism of resistance to third generation EGFRi, we sought to include a model of isogenic NSCLC cells sensitive or resistant to third generation EGFRi. In this additional cell model, we utilized NSCLC cells from the cell line H1975. The H1975 cell line is resistant to erlotinib due to a T790M mutation that increases the affinity of the EGFR receptor for ATP relative to its affinity to erlotinib. Along with other cell lines harboring T790M mutations, H1975 cells remain sensitive to the third-generation EGFR-inhibitor rociletinib (also referred to as CO-1686). Two rociletinib-resistant (COR) clones derived from H1975 cells, COR1-1 and COR10-1, were included in this study. Investigation of EMT markers by western blot analyses for these cell lines (**Figure 1C**, quantified in **Figure 1F**, corresponding total protein images used for normalization in **Figures S1A, B**) were performed. We found that, like the mesenchymal-like erlotinib-resistant cell lines derived from HCC827, the COR1-1 and COR10-1 rociletinib-resistant cells derived from H1975 cells, displayed a reduction in CDH1 (E-cadherin), a moderate upregulation of CDH2 (N-cadherin), and a prominent upregulation of VIM (vimentin) compared to the H1975 parental cells (**Figures 1C, F**).

### Epithelial Phenotype Correlated With High Spheroid Forming Capacity in Homotypic 3D Spheroid Cultures

**Figure 2:**
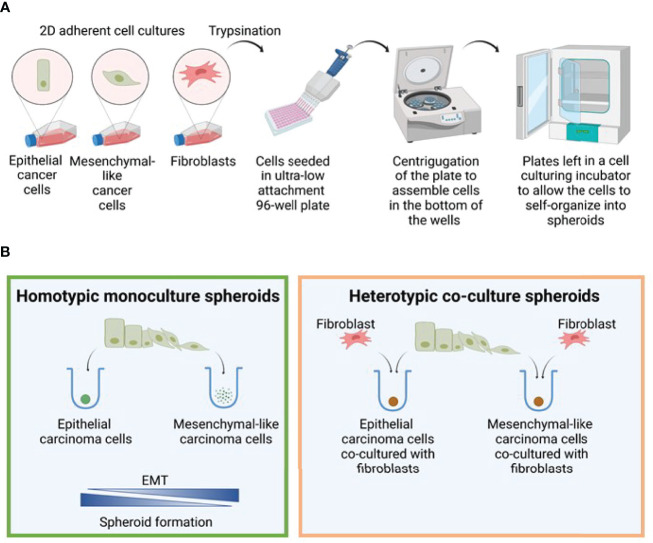
Spheroid formation model. **(A)** Model figure depicting the generation of 3D monoculture spheroids in round bottom ultra-low attachment plates. **(B)** The efficiency of spheroid formation is closely linked to the degree of EMT, and the epithelial cells generated spheroids in monoculture much more efficiently than the mesenchymal cells. In contrast, both epithelial and mesenchymal phenotypes were able to form spheroids when co-cultured with fibroblasts. Figure created with biorender.com.

**Figure 3:**
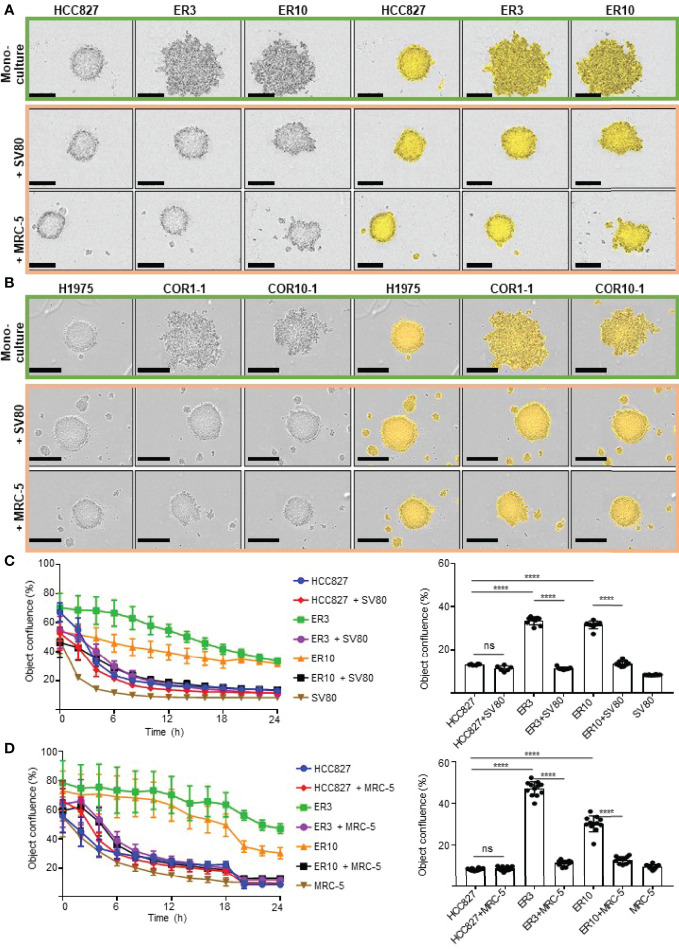
Monitoring cell aggregation and 3D spheroid formation ability in real time using the IncuCyte live cell imaging system. **(A)** The lung adenocarcinoma cell line HCC827 and erlotinib-resistant sub-clones of this cell line (ER3, ER10) were cultured as homotypic spheroids (upper panel) and in combination with lung fibroblast cell lines SV80 (middle panel) or MRC-5 (lower panel). Images were obtained with the IncuCyte live cell imaging system 24 h after seeding in the ultra-low attachment (3D) 96-well plates. The IncuCyte confluence mask (yellow) was generated for the quantification shown in **(C, D)**. **(B)** The NSCLC cell line H1975 and rociletinib-resistant sub-clones (COR1-1 and COR10-1) were cultured as homotypic spheroids (upper panel) or in combination with lung fibroblast cell lines SV80 (middle panel) or MRC-5 (lower panel). Images were obtained with the IncuCyte live cell imaging system 24 h after seeding in the ultra-low attachment (3D) 96-well plates. Spheroid formation efficiency was measured by quantification of the object confluence measured by the IncuCyte Zoom microscope and software-generated confluence mask (yellow) in HCC827, ER3, or ER10 cell monocultured spheroids or as heterotypic co-culture spheroids together with **(C)** SV80 or **(D)** MRC-5. Object confluence over the 24 h time-course and the values for the 24 h endpoint is given. Spheroid formation assays were repeated at least three times, and representative results from one experiment with n = 6-10 technical replicates are presented as mean +/- SD. One-way ANOVA followed by Tukey’s multiple comparisons test was performed to calculate statistical differences in object confluence at 24 h. ****P ≤ 0.0001. ns, not significant.

From the cell line models characterized above, we aimed to generate a 3D cell mono- and co-culture system comprising NSCLC cells of various phenotypes and fibroblast cell lines SV80 or MRC-5. The method for spheroid generation was adapted from Amann et al. (67), and described schematically in **Figure 2A**. First, to test the impact of epithelial-mesenchymal phenotype on the spheroid formation capacity of the generated cell line clones, we tested the ability of the various clones to aggregate and generate multicellular 3D spheroids in monoculture (**Figure 2B**, left), and next as heterotypic co-culture spheroids (**Figure 2B**, right). Briefly, for both monoculture and co-culture spheroids the seeded cells were monitored as they self-aggregated in the wells of U-shaped ultra-low attachment 96-well plates. Phase-contrast images obtained every second hour by the IncuCyte platform revealed that the epithelial HCC827 cells formed compact 3D spheroids when aggregated as a monoculture (**Figure 3A**). In contrast, the mesenchymal-like ER cells (ER3 and ER10 clones) did not generate tight spheres and only loosely adhered to each other in ‘grape-like’ structures (**Figure 3A**). As described above, the ER20 and ER30 cells do not display the same degree of mesenchymal phenotype as ER3 and ER10, and the ER20 and ER30 cells were able to form more tightly packed spheroids than the mesenchymal sub-clones (ER3 and ER10) in monoculture (**Figure S2A**).

A comparable pattern of aggregation and spheroid formation was observed in the H1975 cell model, where the mesenchymal-like EGFRi-resistant sub-clones COR1-1 and COR10-1 remained as ‘grape-like’ aggregates of cells and did not form compact spheroid structures in monoculture, as observed in the epithelial parental H1975 monocultures (**Figure 3B** and **S2B**). Spheroid formation in monoculture was inversely correlated with the extent of the mesenchymal phenotype in HCC827 and H1975 cells with corresponding resistant clones (**Figures 2B**, **3A–D**, **S2A, B**).

### Co-Culture With Fibroblasts Facilitated Spheroid Formation in Carcinoma Cells With Mesenchymal-Like Phenotypes

Next, we aimed to evaluate if co-culture with fibroblasts could allow a more efficient generation of multicellular spheroids and enable studies of both epithelial and mesenchymal-like cells in physiologically relevant heterotypic 3D cultures (**Figure 2B**, right). We found that the HCC827 and H1975 parental cell lines and all EGFRi-resistant clones derived from these cells were able to form compact heterotypic spheroids when seeded as co-cultures together with the lung fibroblast cell lines SV80 or MRC-5 (**Figures 2B**, **3A, B**). Thus, while epithelial cells were also able to form spheroids in monoculture, only co-culture with fibroblasts facilitated formation of compact spheroids in the mesenchymal-like phenotypes. To conclude, by introducing fibroblasts to the culture, the study of carcinoma cells of both epithelial and mesenchymal phenotypes in more physiologically relevant 3D cultures was enabled.

### The Impact of Epithelial-Mesenchymal Phenotype and Fibroblast Co-Culture on Spheroid Formation Dynamics

To gain more insight into the dynamics of spheroid formation from mono- and co-culture cell aggregates, we monitored the spheroid formation in real-time by obtaining phase-contrast images of the cultures every two hours. The IncuCyte Zoom microscope system was applied for live-cell imaging in this study. Using the built-in IncuCyte Zoom software, a confluence mask was generated for each image (**Figures 3A, B**). The confluency mask, visualized in yellow in the images on the right side of **Figures 3A, B**, was applied to quantify the object confluence, which was used as a surrogate marker of spheroid formation ability in this study. Time-lapse IncuCyte imaging and quantification of object confluence (**Figures 3C, D**) showed that the mesenchymal clones ER3 and ER10 formed compact spheroid structures with similar kinetics as epithelial HCC827 cells in co-culture with SV80 or MRC-5 fibroblasts. This result confirms the results observed by visual inspection of images in **Figures 3A, B**.

### Histology and Tissue Organization of the 3D Heterotypic Spheroids

**Figure 4:**
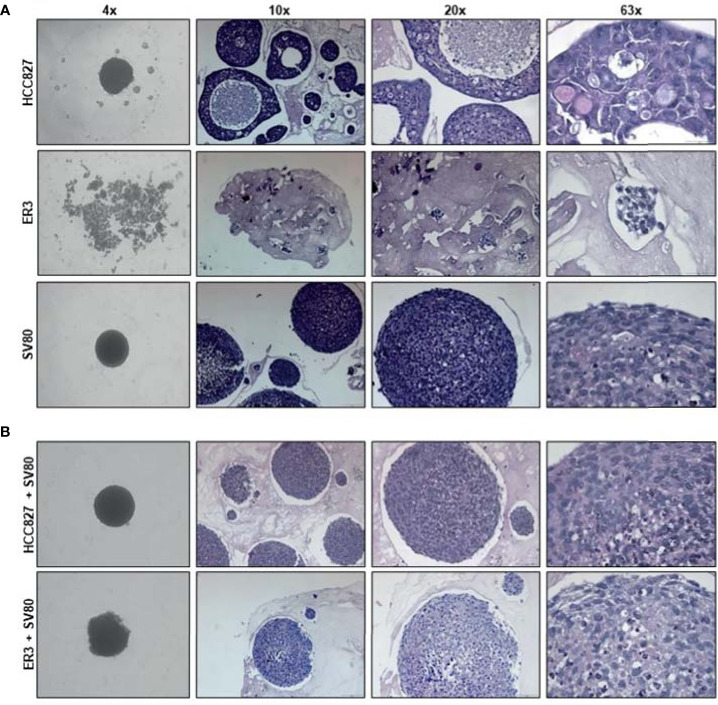
Histological characteristics of homotypic and heterotypic co-culture spheroids. **(A)** Monoculture spheroids of HCC827 parental cells, the erlotinib-resistant clone ER3, and the fibroblast cell line SV80 were cultured for seven days in ultra-low attachment plates before fixation, paraffin embedding, and sectioning. Spheroids are stained with hematoxylin and eosin (H&E). ER3 cells formed only loosely attached clusters of cells captured in a thrombin gel before paraffin embedding. **(B)** Heterotypic co-culture spheroids consisting of SV80 fibroblasts and HCC827 parental or ER3 cells in a 2:1 ratio. Spheroids were cultured for seven days in ultra-low attachment plates before fixation, paraffin embedding, and sectioning. Spheroids are stained with H&E. The light pink matrix surrounding the loosely attached cell clusters of ER cells, and the spheroids is the serum-thrombin clot used to cast the spheroids. Magnification is indicated.

As co-culture with fibroblasts also allowed for spheroid formation by the mesenchymal-like clones, we decided to further characterize the spheroid models made by the parental epithelial cell line HCC827, or the mesenchymal-like erlotinib-resistant clone ER3, cultured alone or in combination with the SV80 fibroblast cell line. To characterize the morphology of the generated homotypic and heterotypic spheroids at the microscopic level, the spheroids were formalin-fixed and embedded in paraffin. H&E staining of the paraffin sections was performed to observe the histology of the spheroids (**Figure 4**). At the microscopic level, it is evident that the mesenchymal ER3 cells cannot form solid spheroids in monoculture, and the ER3 cells remain only loosely attached as single-cell or as smaller ‘grape-like’ aggregates, as observed scattered throughout the casting matrix used for collection and embedding (**Figure 4A**). The HCC827 parental cells, on the other hand, grouped together to form a thick layer of disorganized cancer cells (**Figure 4A**). The fibroblasts in monoculture formed solid compact homotypic spheroids. Upon heterotypic co-culture with SV80 fibroblasts, both HCC827 parental cells and ER3 cells formed compact spheroids (**Figure 4B**). For HCC827 and SV80 monoculture spheroids, as well as both heterotypic spheroids, central areas of hypoxia could be observed in a majority of the spheroids.

### Intra-Spheroid Organization of Epithelial and Mesenchymal Carcinoma Cells When Co-Cultured With Fibroblasts

**Figure 5:**
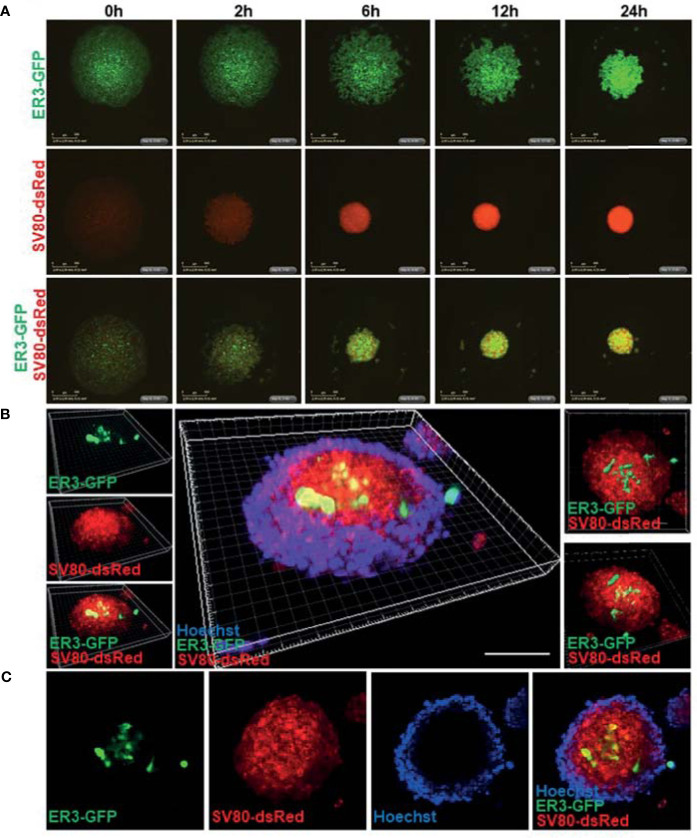
Real-time spheroid formation and compound penetration in the 3D heterotypic co-culture spheroids made of fluorescent transgene expressing cells. **(A)** HCC827 ER3 cells and SV80 fibroblast cells were stably transduced by lentiviral particles harboring the GFP and dsRed transgene, respectively. Transduced cells were subsequently sorted by FACS to obtain a population of cells with a uniform transgene expression. 3D spheroid formation was studied by time-lapse imaging using the IncuCyte system. Images were obtained every 2 h using 4x objective. Representative images from the 0-24 h time interval are shown for homotypic ER3 and SV80 cells, as well as the ER3 and SV80 heterotypic spheroids. Scalebar = 500 μm. **(B)** The illustration shows a 3D heterotypic co-culture spheroid of HCC827ER3 (GFP) and SV80 (dsRed) counterstained by Hoechst (blue). Images obtained by Zeiss confocal microscope. Z-stack depth = 71.77 µm. Reconstruction by IMARIS software. Scale bar: 100 µm. **(C)** ER3 and SV80 heterotypic spheroids were stained for 7 days with Hoechst to visualize the penetration of drugs of comparable size.

To enable a simple and reliable model system where various cell types in the co-culture system could be easily distinguished, the HCC827, ER3, and ER10 cancer cells were transduced by lentiviral vectors harboring GFP and the SV80 fibroblasts were transduced with lentiviral vectors harboring dsRed fluorescent protein. The transduced cells were sorted by FACS based on the expression of the fluorescent transgene to obtain a homogenous population of transgene expressing cells. Subsequent time-lapse fluorescence imaging by IncuCyte Zoom microscope was applied to explore in real time the ability of ER3 and SV80 cells to aggregate to form spheroid structures (**Figure 5A**). The dynamics of spheroid formation from cell aggregates was further characterized by videos capturing the process (**Videos S1** **–** **S3**). These experiments confirmed that the ER3 GFP-labeled cells do not form solid spheroids and are still only loosely attached in grape-like structures 24 h post-seeding (**Figure 5A** and **Video S1**). The SV80 fibroblasts, on the other hand, readily formed compact round multicellular structures, and as soon as six hours post-seeding, a red fluorescent signal was detected from solid spherical structures with a well-defined border (**Figure 5A** and **Video S2**). We obtained comparable results with another fibroblast cell line, MRC-5 (data not shown). The co-culture aggregates containing the ER3 GFP-labeled cells and the SV80 dsRed-labeled fibroblasts also formed compact spheroids within six hours post-seeding (**Figure 5A**, **Video S3**).

To further characterize the inter-cellular structure of the heterotypic spheroids, spheroids formed by fluorescent cells were harvested 10-days post-seeding and stained with Hoechst for visualization of nuclei by confocal microscopy (**Figure 5B**). A 3D projection was generated from the confocal images using the IMARIS software. This 3D z-stack reconstruction showed that in the heterotypic co-culture spheroids consisting of GFP-positive ER3 cells and dsRed positive SV80 cells, the mesenchymal-like ER3 cancer cells tend to be localized inside the spheroid surrounded by the fibroblasts. Furthermore, we observed that the Hoechst nuclear dye (MW 452.6) could not penetrate the whole structure, and the Hoechst stain is therefore only seen at the outer rim of the spheroid (**Figures 5B, C**). An animation of the 3D projection (**Figure 5B**) made in IMARIS is shown in **Video S4**.

### *In-Depth* Characterization of Mono- and Co-Culture Spheroids Using Immunohistochemistry and Imaging Mass Cytometry

**Figure 6:**
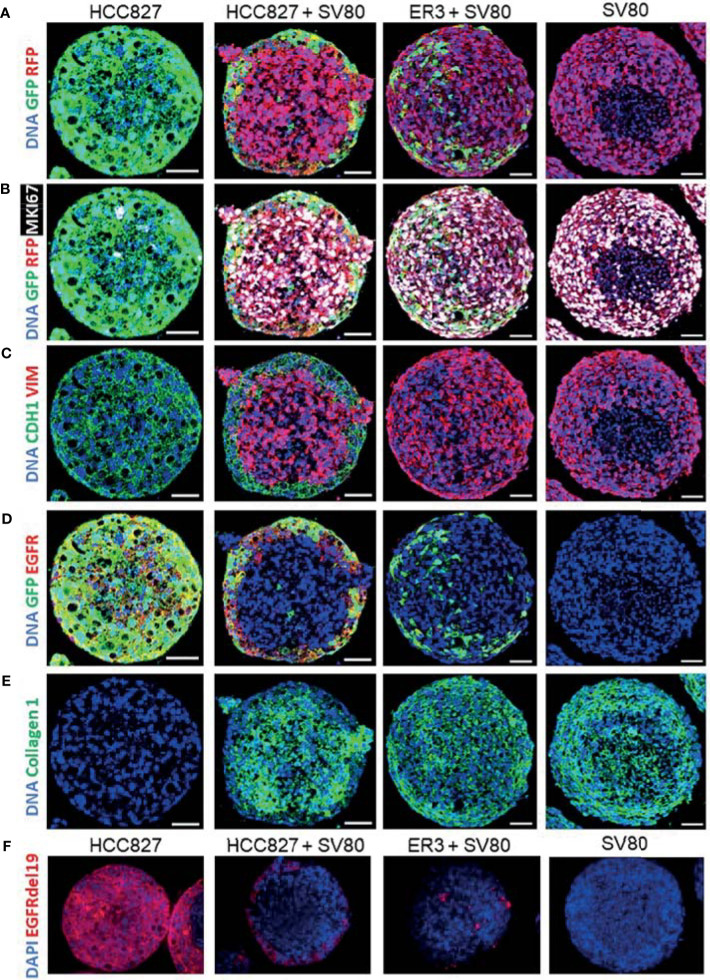
Characterization of spheroids using immunohistochemistry with fluorescent detection and imaging mass cytometry. HCC827-GFP monoculture, HCC827-GFP + SV80-dsRed co-culture, ER3-GFP + SV80-dsRed co-culture, and SV80-dsRed monoculture spheroids were stained with an imaging mass cytometry panel of 14 heavy metal-tagged antibodies (**Table 3**). For each condition, five ROIs containing a single spheroid were ablated by a Hyperion imaging mass cytometer (Fluidigm, Inc.). Representative ROIs are displayed showing **(A)** GFP and RFP, **(B)** GFP, RFP and MKI67 (Ki67), **(C)** CDH1 (E-cadherin), and VIM (Vimentin), **(D)** GFP and EGFR, **(E)** and Collagen 1. DNA staining displayed in all images is a combination of Iridium intercalator stain (Ir191 and Ir193) and Histone H3. Images were pseudo-colored in MCD viewer software (Fluidigm) to enable visualization of multiple channels per ROI, and for each channel the minimum and maximum intensity display settings are manually set to be kept constant between the samples. Scalebar = 50 µm **(F)** Immunohistochemistry staining with the EGFRdel19 mutation-specific antibody and AF647 fluorescence tagged secondary antibody together with DAPI counterstain. Fluorescent images were taken with a Zeiss Collibri7 fluorescence microscope.

For in-depth molecular analysis, an imaging mass cytometry antibody panel containing 14 heavy metal-tagged antibodies (**Table 3**) was developed. The panel includes markers of EMT, proliferation, and extracellular matrix proteins. Antibodies against GFP and RFP were included in the panel to allow efficient and endogenous marker-independent separation of the cell types in the images for further downstream analysis. For each condition, five ROIs containing a single spheroid were ablated with a Hyperion imaging mass cytometer, and representative pseudo-colored images of selected markers from the panel are displayed in **Figures 6A–E**. The GFP and RFP staining confirmed that ER3 cells were found scattered in between the SV80 fibroblast cells in the heterotypic ER3-SV80 co-culture spheroid. In contrast, for the heterotypic HCC827-SV80 spheroids, a different organization and a more prominent self-sorting of the cells was observed. The HCC827 cells were localized towards the edge (cortex) of the spheroid when co-cultured with the fibroblasts, and the fibroblasts were found in the center (medulla) of the spheroid (**Figure 6A**). The proliferation marker MKI67 (Ki67), visualized together with GFP and RFP, indicated that the SV80 fibroblasts are highly proliferative (**Figure 6B**). The organization of the heterotypic HCC827-SV80 spheroids was also confirmed by CDH1 (E-cadherin) and VIM (vimentin) staining, as VIM (vimentin) is only expressed in the mesenchymal SV80 fibroblasts while CDH1 (E-cadherin) is only expressed in the HCC827 cells (**Figure 6C**). We also observed EGFR expression only in the mono- and co-culture spheroids containing HCC827 or ER3 cells and not in the SV80 monoculture spheroids (**Figure 6D**). In contrast, collagen type 1 was only observed in SV80-containing spheroids (**Figure 6E**), indicating that the fibroblasts are the sole cell type responsible for the observed collagen deposits in this model system. The organization of the cells within the heterotypic spheroids of both HCC827 and ER3 when co-cultured with SV80 fibroblasts was also confirmed by IHC-P/IF using an antibody explicitly targeting the EGFRdel19 mutation present in the HCC827 parental cell line and conserved in the ER3 cells (**Figure 6F**).

**Figure 7:**
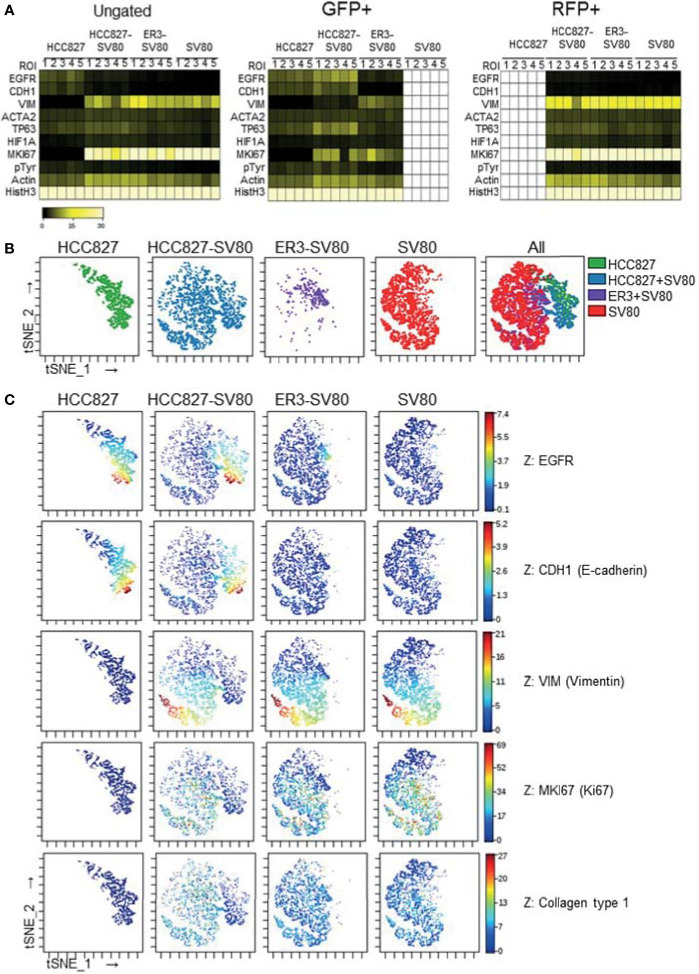
In-depth analysis of imaging mass cytometry single-cell data from mono- and co-culture spheroids. **(A)** Single-cell expression data obtained from segmentation of the imaging mass cytometry experiment are displayed as a heatmap of the ungated (left), GFP+ (middle) and RFP+ (right) populations. Single-cell data was first generated as the mean pixel intensity for each cell, and the median intensity of all cells within a given population is displayed in the heatmaps. **(B)** The tSNE algorithm was applied on the ungated population based upon expression of the three markers EGFR, CDH1 (E-cadherin), and VIM (vimentin). viSNE plots displaying the distribution of cells from the different samples. **(C)** Marker expression of EGFR, CDH1 (E-cadherin), VIM (vimentin), MKI67 (Ki67), and Collagen type 1 displayed on the viSNE plots.

Single-cell expression data was generated by segmenting the images generated by IMC based on nuclei and GFP/RFP marker expression. The single-cell data were then gated based upon GFP and RFP transgene expression (**Figure S3**) and the median fluorescence intensity of the remaining markers was visualized in heatmaps for the ungated, GFP+ and RFP+ populations across the different spheroid groups (**Figures 7A** and **S4**, median fluorescence intensity values listed in **Figure S4**). The tSNE algorithm was further applied on the ungated population based upon expression of the three markers: EGFR, CDH1 (E-cadherin), and VIM (vimentin). Visualization of the tSNE parameters (viSNE) displaying the distribution of cells from the different samples is shown in **Figure 7B**. HCC827 cells were well separated from the ER3 and SV80 cells on the viSNE plot, while the mesenchymal ER3 cells and SV80 fibroblasts were partially overlapping. The expression of EGFR, CDH1 (E-cadherin), VIM (vimentin), MKI67 (Ki67), and collagen type 1 is visualized in **Figure 7C** and the remaining markers in **Figure S5**.

### Drug Response and *InDepth* Characterization of Erlotinib-Treated Co-Culture Spheroids

**Figure 8:**
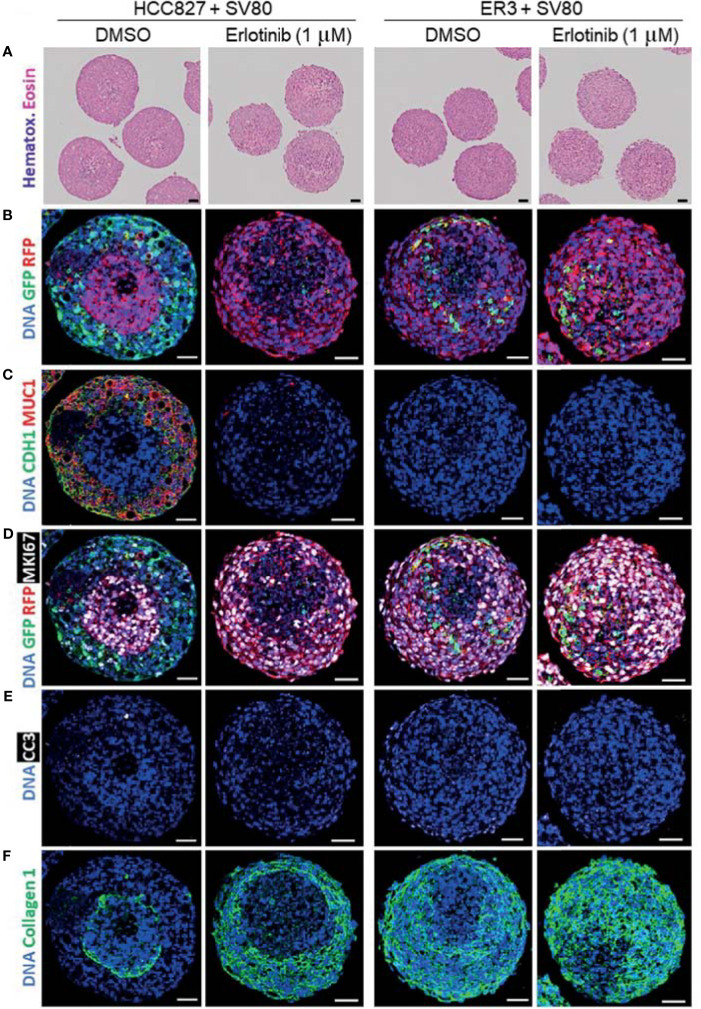
Characterization of erlotinib-treated heterotypic spheroids using H&E staining and imaging mass cytometry. **(A)** H&E-stained sections of heterotypic HCC827-GFP + SV80-dsRed co-culture, ER3-GFP + SV80-dsRed co-culture spheroids. **(B–F)** Paraffin sections of vehicle (DMSO) and erlotinib- treated heterotypic HCC827-GFP + SV80-dsRed co-culture, ER3-GFP + SV80-dsRed co-culture spheroids were stained with an imaging mass cytometry panel of 19 heavy metal-tagged antibodies (**Table 3**). For each condition, five ROIs containing a single spheroid were ablated by a Hyperion imaging mass cytometer (Fluidigm, Inc.). Representative ROIs are displayed showing: **(B)** GFP and RFP, **(C)** epithelial marker CDH1 (E-cadherin) and MUC1 (Mucin1/CD227), **(D)** GFP, RFP and proliferation marker MKI67 (Ki67), **(E)** apoptosis marker Cleaved caspase 3 (CC3), and **(F)** Collagen 1. DNA staining by Iridium intercalator stain (Ir191 and Ir193). Images were pseudo-colored in MCD viewer software (Fluidigm) to enable visualization of multiple channels per ROI, and for each channel the minimum and maximum intensity display settings are kept constant between the samples. Scalebar = 50 µm.

As a *proof-of-principle* to show that the spheroid model utilized in this study is applicable for drug screening experiments, we treated the heterotypic co-culture spheroids consisting of SV80 fibroblasts and either erlotinib-sensitive HCC827 cells or erlotinib-resistant ER3 cells for seven days with either erlotinib (1 µM) or vehicle (DMSO) control. The spheroids were further formalin-fixed and embedded in paraffin. H&E staining of the paraffin sections revealed the histology of the treated spheroids (**Figure 8A**).

An imaging mass cytometry antibody panel containing 19 heavy metal-tagged antibodies (**Table 3**) was applied for in-depth molecular analysis of the treated spheroids. Representative pseudo-colored images of selected markers from the panel are displayed in **Figures 8B–F**. By examining the GFP and RFP staining, we observed significant disappearance of erlotinib-sensitive HCC827 (GFP positive) cells within the HCC827-SV80 co-culture spheroids upon erlotinib treatment (**Figure 8B**). On the other hand, the presence of erlotinib-resistant ER3 cells appeared intact in the erlotinib treated ER3-SV80 co-cultures (**Figure 8B**). Also, CDH1 (E-cadherin) and MUC1 (Mucin1/CD227), both markers that are expressed by the epithelial HCC827 cells, were absent in the erlotinib treated HCC827-SV80 spheroids (**Figure 8C**). By investigating MKI67 (Ki67) expression in the co-cultures, it was revealed that the erlotinib treatment did not impair the proliferative phenotype of the SV80 fibroblast (RFP positive) cell line in either of the spheroid co-culture systems (**Figure 8D**), and the cleaved caspase 3 (CC3) expression indicates that little or no apoptosis was induced in the SV80 cells upon erlotinib treatment (**Figure 8E**). Further, a substantial reorganization of the ECM (collagen type 1) was observed in the HCC827-SV80 co-cultures treated with erlotinib (**Figure 8F**), as the population of HCC827 cells diminished and SV80 cells expanded. In contrast, no major reorganization of the ECM was observed in the ER-SV80 co-cultures.

**Figure 9:**
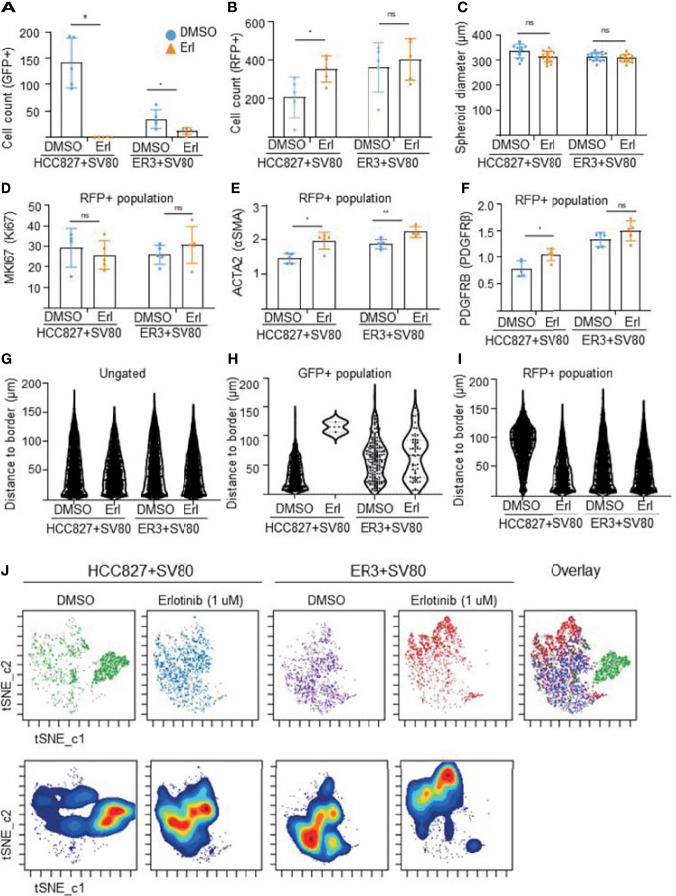
In-depth analysis of imaging mass cytometry data from erlotinib treated spheroids. Absolute cell counts for HCC827+SV80 and ER3+SV80 heterotypic spheroids treated with either vehicle control (DMSO) or erlotinib, respectively. Absolute cell counts for **(A)** GFP+ cells (cancer cells), and **(B)** RFP+ cells (fibroblasts) is shown. Individual values represent median expression of each ROIs, and the mean +/-SD is plotted. Statistical significance in absolute cell counts were calculated in cytobank using the Mann-Whithey U-test **(C)** The diameter of erlotinib treated versus vehicle control (DMSO) treated spheroids measured by quantifying H&E-stained images using the measuring tool in Fiji. Mean diameter +/- SD for each condition is shown, and the statistics is performed in GraphPad Prism using the Mann-Whithey U-test. **(D–F)** Median expression of proliferation marker MKI67 (Ki67) in the nuclei of the RFP+ population **(D)**. Median expression of ACTA2 (αSMA) **(E)**, and PDGFRB (PDGFRβ) **(F)** in the RFP+ (fibroblast) populations of HCC827+SV80 and ER3+SV80 heterotypic spheroids treated with either erlotinib or vehicle (DMSO) control. Individual values represent median expression for each ROIs, and the mean +/- SD is plotted. Statistical significance in channel expression were calculated in cytobank using the Mann-Whithey U-test **(G–I)** Distance to border measurements displayed as Violin plots for the **(G)** ungated (all) population, **(H)** GFP+ population, and **(I)** RFP+ population. **(J)** The tSNE algorithm was applied on the ungated population based upon expression of the markers EGFR, CDH1 (E-cadherin), VIM (vimentin), MET (c-Met), pan-cytokeratin, GFP and RFP. viSNE plots displaying the distribution of cells from the different samples. NS = P > 0.05, *P ≤ 0.05, **P ≤ 0.01.

The visual evaluation of drug response to erlotinib in the HCC827 and ER3 cells when co-cultured with SV80 was also confirmed and quantified by gating the GFP+ and RFP+ cell populations (**Figure S6**) and quantifying the number of GFP+ cells within the treated spheroids (**Figure 9A**). As expected from visual inspection, the quantification confirmed that the majority of HCC827 cells did not survive the erlotinib treatment as the number of GFP+ (HCC827) cells decreased dramatically (from 22% to 0.8%) in the HCC827-SV80 spheroids. Less reduction in the number of GFP+ (ER3) cells was observed in the ER3-SV80 spheroids decrease from 4.6% to 2.0% upon erlotinib treatment). In contrast, the number of RFP+ (SV80) cells increased in both co-culture systems, although the increase was only statistically significant in the HCC827-SV80 spheroids (**Figure 9B**). In accordance with this, we observed no significant difference in the size of the spheroids after erlotinib treatment as measured by the spheroid diameter (**Figure 9C**).

Quantification of marker expression within the RFP+ (SV80) population revealed no significant change in nuclear MKI67 (Ki67) expression in the co-cultures upon treatment (**Figure 9D**), further supporting that toxicity is not induced in the fibroblasts upon erlotinib treatment. This was also confirmed by no statistically significant differences in the percentage of MKI67 (Ki67) or phophso-Histone H3 positive cells in either of the populations upon treatment (**Figure S7**). In the RFP+ population (SV80 cells), we also observed increased median expression of ACTA2 (alpha smooth muscle actin, αSMA) (**Figure 9E**) and PDGFRB (platelet derived growth factor receptor beta, PDGFRβ) (**Figure 9F**) after erlotinib treatment, indicative of a more CAF-like phenotype. Nuclear HIF1A (hypoxia inducible factor 1 subunit alpha, HIF1α) was also upregulated in the RFP+ population of ER3+SV80 spheroids upon erlotinib treatment (**Figures S8A, B**). No statistically significant differences in the DNA staining by Iridium intercalator (Ir191 and 193) were observed in the nuclei upon treatment at the single cell level in either of the spheroid types included (**Figure S8C**). Heatmaps showing the mean channel intensity of all markers in the ungated, GFP+ and RFP+ populations are shown in **Figure S9**.

To quantify intra-spheroid cell type composition, we used the “distance-to-border” parameter as a quantitative measurement of the position of the cells within the spheroid in the ungated (**Figure 9G**), GFP+ (**Figure 9H**) and RFP+ (**Figure 9I**) populations within the HCC827+SV80 and ER3+SV80 spheroids treated with erlotinib or vehicle (DMSO) control. These violin plots clearly demonstrate the re-organization of the HCC827-SV80 spheroids with a prominent self-sorting upon erlotinib-treatment as the very few surviving GFP+ (HCC827 cells) are detected in a protective location towards the middle of the spheroid (high distance-to-border value) and the average distance-to-border of the RFP+ (SV80) cells decreased as the fibroblast composition changed from centrally localized to diffuse and dominant. The tSNE algorithm was again applied on the ungated population. viSNE plots displaying the distribution of cells from the different samples is shown in **Figure 9J**. Again, the HCC827 cells were well separated from the ER3 and SV80 cells on the viSNE plot, while the mesenchymal ER3 cells and SV80 fibroblasts were partially overlapping. It is also apparent from the viSNE plot that the majority of the HCC827 cells disappear upon erlotinib treatment. The channel expression of the various markers is visualized on the viSNE plots in **Figure S10**.

## Discussion

Despite the impact of the TME and dynamic interactions between cancer cells and stromal cells on cancer progression and treatment efficacy, the most widely used *in vitro* pre-clinical cancer model remains 2D cell cultures. Certainly, the flat biology and the homogenous nature of cell line-based models are far from sufficient to recapitulate the complex architecture and cellular diversity of cancers *in situ.* In fact, the widespread application of insufficient pre-clinical models has been suggested as one of the main reasons why promising anti-cancer drugs fail in the translation from lab to clinic. To reduce this translational gap, we have developed and characterized a 3D heterotypic co-culture model comprising NSCLC cells of varying epithelial-mesenchymal phenotypes with fibroblasts. To measure the effects of clinically relevant targeted therapy, NSCLC cells sensitive or resistant to EGFRi were included. Spheroids represent several important tumor characteristics, including 3D geometry, chemical and physical gradients, and cell-cell interactions. Despite this complexity, spheroid models are relatively easy to work with and compatible with high-throughput drug screenings. By combining spheroid models with techniques such as high-dimensional imaging, this model holds a significant potential for studies of cancer-stroma interactions and therapy responses and may also serve as a good model to optimize antibody panels and segmentation strategies prior to their application on more complex tissues. This model may be further improved by modifying the type of fibroblasts used in the co-cultures to enable comparisons between normal pulmonary fibroblasts and CAFs isolated from NSCLC tissue, or by including other cells of the TME such as immune cells to obtain a more relevant model for *in vitro* screening of novel cancer therapies and studies of complex mechanisms of resistance in heterotypic cancers.

In two cell-line models based on NSCLC HCC827 and H1975 parental cells and their respective erlotinib- or rociletinib-resistant clones, we observed a shift towards a more mesenchymal-like phenotype in multiple EGFRi-resistant clones (54, 55, 66). In the HCC827 model, loss of the characteristic cobblestone epithelial morphology, downregulation of *CDH1* (E-cadherin), and upregulation of the mesenchymal markers *CDH2* (N-cadherin) and *VIM* (vimentin) were prominent at both mRNA and protein levels in the erlotinib-resistant clones ER3 and ER10. Similarly, in the H1975 model, increased expression of CDH2 (N-cadherin) and VIM (vimentin) and decreased expression CDH1 (E-cadherin) were observed in the third generation EGFRi (rociletinib)-resistant subclones. These observations are consistent with previous descriptions of the HCC827 and H1975 cell models (54, 66, 68). These findings also support studies from our laboratory and others that have established EMT as a mediator of acquired drug resistance to EGFR targeted therapies in NSCLC (54, 66, 69–71). EMT and upregulation of AXL has further been associated with resistance to other NSCLC therapies, including cytotoxic therapies, immune checkpoint inhibition, and immune cell-mediated killing (5, 72–75).

In the 3D spheroid model, EGFRi-sensitive epithelial cells readily aggregated and formed compact spheroids in monoculture, while the resistant cells with mesenchymal-like phenotypes (ER3, ER10, COR1-1, and COR10-1) were dependent on co-culture interactions with fibroblasts to form compact spheroids. Interestingly, the spheroid formation capacity seemed to be further linked to the degree of EMT, as observed in the most mesenchymal ER3 and ER10 cells, compared to the less mesenchymal ER20 and ER30 cells. In contrast to ER3 and ER10, the ER20 and ER30 cells displayed less prominent expression of markers of a mesenchymal phenotype and were also able to form more compact spheroid structures than ER3 and ER10 after 24 hours in monoculture. We have previously observed reduced spheroid-formation capacity when EMT was induced in the mammary epithelial cell line MCF10A by overexpression of the EMT transcription factors TWIST or SNAI2 (76). Taken together, these findings indicate an inverse relationship between the mesenchymal features developed as a resistance mechanism against EGFR inhibitors in this model system and the ability to form compact multicellular spheroids from cell aggregates. This finding might appear counterintuitive because a more mesenchymal or an intermediate E/M phenotype in particular, is also associated with cancer stem cell properties and increased tumor-initiating potential (10, 35, 77, 78). However, our current observation, which describes self-aggregation and self-sorting abilities rather than proliferation and sphere-formation potential, could be explained by mesenchymal cells being more motile and lacking the strong cellular adhesion properties found in epithelial cells. These features make the mesenchymal cancer cells less prone to generate strong inter-cellular adhesions and organize into compact spheroid structures. The fibroblasts also display a greater ability to generate compact spheroid structures compared to the mesenchymal ER3 cells, even though they exhibit comparable expression of the cell adhesion molecules evaluated in this study. This indicates that additional adhesion systems, including secretion of ECM proteins like fibronectin, and expression of integrins like alpha5beta1 integrin or other factors, may be responsible for the increased spheroid generation ability of fibroblasts compared to ER3 cells. The fibroblasts in 3D culture further showed a prominent collagen deposition that may support their superior spheroid formation ability compared to the mesenchymal ER3 cells. The differences were even more prominent from the GFP and RFP staining in the live-cell imaging and the IMC staining. The cancer cells in the ER3-SV80 heterotypic co-culture spheroids were localized as single cells or smaller islands towards the spheroid center and surrounded by fibroblasts. In contrast, the HCC827 parental cells in co-culture with fibroblasts displayed strong self-sorting and were localized towards the edge of the co-culture spheroid surrounding a core of fibroblasts. A plausible explanation for this observation may be that the mesenchymal-like EGFRi-resistant cancer cells are more adapted to survive within a hypoxic microenvironment, compared to the parental cells that are expected to be less adaptive to microenvironmental changes, including the expected lack of oxygen as well as nutrients in the core of the spheroid (79). The ability to generate spheroids in monoculture and the observed differences in the cellular organization of the co-culture spheroids between the epithelial and mesenchymal phenotypes are also likely to be dependent on their differences in expression of cellular adhesion molecules (CAMs). When self-aggregation is followed by the various cell types organizing themselves into a specific pattern of segregation, this can be referred to as self-sorting (43). Friedlander et al. observed that murine sarcoma cells transfected with *CDH2* (N-cadherin) or liver cell adhesion molecule (L-CAM), a molecule structurally related to N-cadherin, aggregated more rapidly than isogenic cells not expressing these cellular adhesion molecules (80). Cells expressing high levels of L-CAM or CDH2 (N-cadherin) also self-sorted together with cells expressing the same CAM, and this segregation was inhibited by antibodies specifically targeting the transfected CAM (80). Furthermore, Steinberg and colleagues demonstrated that differential expression of the cell adhesion molecule CDH3 (P-cadherin) in embryonic cells not only segregated cells with different CDH3 (P-cadherin) levels from each other, but also caused the less cohesive cells to envelop a core of more cohesive cells (48). When the cells were mixed, they formed a “sphere within a sphere” configuration with the cell population expressing the most CDH3 (P-cadherin) forming islands within the sphere. In addition, for systems containing ECM components, cell-ECM interactions and integrin expression can also contribute to the cellular organization (81). A similar self-sorting pattern to ours with the epithelial cancer cells in the outer cortex layer of the sphere and the fibroblasts in the medulla has also been observed in other heterotypic spheroid cultures (45). Whether or not the cancer cells sort into different compartments or intermix depends in part on the balance between the binding forces from the cell-to-cell interactions and the interactions between the cancer cells and the various components of the TME including the various ECM components (82). Since the mesenchymal-like ER3 cells display a greater phenotypic similarity to the mesenchymal SV80 fibroblasts than the epithelial HCC827 cells, this could possibly explain that also a higher degree of segregation between the two cell types were observed in the HCC827 co-culture compared to the ER3 co-cultures. In addition, we observed prominent collagen deposition by the SV80 cells, and it is therefore likely that cell-ECM and heterotypic cell-cell interactions play a vital role in the intra-spheroid organization in our model system.

To allow a high-dimensional characterization of the 3D models, IMC antibody panels containing 14-19 heavy metal-tagged antibodies were designed. These panels included markers of EMT, proliferation, apoptosis and ECM components, as well as GFP and RFP to differentiate the fluorescently labeled cell types in the co-cultures for downstream analysis. To quantify expression of the various protein markers at a single-cell level, cells were segmented based on their nuclei and cytoplasmic staining and the mean pixel intensity of each marker was measured for each cell in the segmentation mask from the corresponding multiplexed IMC images. Our first aim was to separate the cancer cells and fibroblasts based upon their GFP and RFP expression. We therefore gated the GFP+ and RFP+ populations, and a strict gating strategy was applied to exclude double-positive cells from the analysis. No RFP positive cells were observed in the HCC827 monoculture samples, and no GFP positive cells were observed in the SV80 monoculture samples. Thus, in this setting, cells positive for both GFP and RFP indicate the inclusion of neighboring cell staining within the predicted shape of those particular cells within the segmentation mask. The median fluorescence intensity of the different markers from the single-cell data was then visualized in heatmaps for the ungated, GFP+ and RFP+ populations. This strategy was also applied to evaluate the success of the single-cell segmentation itself, as more double-positive cells will also be an indicator of sub-optimal cell segmentation. Segmentation methods that were tested included expansion of the nuclei objects identified by DNA-intercalator staining by either 1 or 2 pixels, and an alternative method applying the Mesmer algorithm. Mesmer is a deep learning-enabled segmentation algorithm that utilizes the pre-trained MultiplexSegmentation dataset and user-defined aggregate images of markers representing either nuclei or cytoplasm as input (63). Segmentation masks generated by Mesmer using Histone H3 or Iridium intercalator to identify nuclei and GFP and RFP to identify the cytoplasm were considered the most successful from the tested segmentation strategies and were therefore chose to use segmentation masks generated by Mesmer in the downstream analysis. Furthermore, the tSNE algorithm was also applied on the single-cell data from the ungated population. Interestingly, displaying the markers of EMT on the viSNE plot revealed a remarkable heterogeneity in the expression levels of CDH1 (E-cadherin) in the HCC827 cells and VIM (vimentin) in the SV80 cells that were not visible from the bulk data. Although, the data should be interpreted cautiously as the variation observed in IMC data could also be due to inaccuracies in cell segmentation, previous high-dimensional single-cell data from suspension cultures (66) support the significant E/M heterogeneity of the NSCLC cell lines used in this study.

This study was designed to provide a thorough characterization of multicellular spheroids of isogenic cancer cells of various phenotypes and demonstrate *proof-of-principle* for the applicability of the presented spheroid model to evaluate the impact of cancer cell phenotype in drug screening experiments through high-dimensional and spatially resolved IMC analyses. We treated the heterotypic co-culture spheroids consisting of SV80 fibroblasts and either erlotinib-sensitive HCC827 cells or erlotinib-resistant ER3 cells for seven days with either vehicle control (DMSO) or erlotinib (1 µM). As expected, we observed significant decline in the number of erlotinib-sensitive HCC827 cells within the HCC827-SV80 co-culture spheroids upon erlotinib treatment. Due to the prominent self-sorting of the epithelial HCC827 cells, they were readily exposed to the erlotinib containing medium, while the ER3 cells were positioned in a more protected location towards the hypoxic center of the spheroids. Of note, the model may readily be modified by adjusting the numbers of cells seeded for aggregation, as well as the ratio between cancer cells and fibroblasts. Judah Folkman stated that tumors in the avascular stage remain dormant at diameters of 1 to 2 mm^3^, and further growth is possible only after new capillaries have been formed and the tumors enter the vascular stage (83, 84). The heterotypic spheroid model described in this study had an average diameter of 337 and 315 µm for HCC827 and ER3 containing spheroids, respectively, and a necrotic center were clearly visible. This is in accordance with the seminal papers of tumor spheroid formation from aggregated cells *in vitro*, that state that central necrotic cells were observed occasionally when the spheroids reached a diameter of 200 µm (5 days) and were clearly evident at a diameter of about 300 µm (9 days) (40). Later studies have further demonstrated that the initial growth of a spheroid typically increases exponentially time until a certain value before the tumor spheroid growth decreases and the spheroid size reaches a plateau, typically observed around 400-500 µm (43, 52, 85). Thus, the spheroids contain well-nourished cells in the periphery of the spheroid structure in close contact with the culture medium, dying cells in central necrotic areas of the spheroid structure, where the oxygen and nutrient availability decreases, and cells in a range of intermediate states in the middle of these two extremes (38, 40, 43). The latter cells are more likely to become resistant to radiation and stress because of the altered oxygen tension and cell kinetics (86–88). Hypoxia has further been shown to be an important predictive and prognostic factor in NSCLC and suggested as a major contributor to treatment failure in lung carcinomas (88).

Furthermore, from the 3D projections of intact spheroids generated from confocal microscopy z-stack images, it was evident that the Hoechst (H-33342) DNA dye only stained an outer rim of the spheroid tissues at an approximately 50 µm depth, due to insufficient dye penetration. The limited small molecule tissue penetration is as expected, based on previous reports (89, 90). Hoechst has also previously been suggested as a potential agent for quantifying tissue perfusion, and for allowing selection of cancer cell subpopulations from different areas within tumors or spheroids, as the incomplete Hoechst staining is likely related to stain penetration issues, rather than variable DNA content at low concentrations (89). Thus, the limited staining by Hoechst in the inner spheroid tissues is an important observation since it serves as a surrogate marker for the penetration of drugs into the spheroid. Hoechst has a relatively small molecular weight (MW 452.6), that is comparable to small molecule inhibitors, including erlotinib (MW 393.4) and rociletinib (MW 555.6). Thus, the drug penetration range observed in this model provides a more physiologically relevant penetration with similarities to the drug exposure pattern of carcinomas *in situ.* The ability of the model to provide physiologically relevant drug penetration conditions represents a clear distinction and advantage over the more frequently applied 2D cell culture drug screening models, as the lower drug exposure may also affect the generation and persistence of drug-resistant populations (36, 43, 90). On this note, it is also possible to directly measure the penetration of drugs into tissues with this approach, as has already been demonstrated with platinum chemotherapy (91) and could be readily accomplished for antibody-based therapies using existing heavy-metal conjugation chemistries. At the same time, the erlotinib-resistant cells were also shown to resist treatment in the co-culture model. By investigating MKI67 (Ki67) expression in the co-cultures, it was evident that the SV80 fibroblast cell line preserved its proliferative phenotype upon erlotinib treatment, demonstrating that this model also could be applicable for toxicity testing.

In conclusion, we have established and thoroughly characterized robust pre-clinical models of EGFR inhibitor resistance in NSCLC. We observed an inverse relationship between mesenchymal features developed as a resistance mechanism against EGFR inhibitors and the ability to form compact multicellular spheroids in this model system. However, when co-cultured with human fibroblasts, we were able to generate heterotypic spheroids containing fibroblasts and the most mesenchymal EGFR inhibitor-resistant subclones that generated only loosely attached cell aggregates in 3D monoculture. The spheroid model presented here can be applied to investigate the crosstalk between fibroblasts and cancer cells of various phenotypes in depth and evaluate the impact of cellular phenotype on secreted factors, direct cell-cell interactions, and modulation of ECM components. It took a long time for EMT to be recognized as a potential mechanism for carcinoma progression (13, 15, 17, 18, 92). One of the main reasons for this was the inability to follow the development of human tumors in time and space. Although EMT was traditionally considered a binary process where cells could be either in an epithelial or a mesenchymal state, it is now highly accepted that EMT is not an on/off switch. The process of EMT is rather dynamic over time, and intermediate E/M states exist in normal and malignant cells, making cells with a high degree of plasticity able to move back and forth across the EMT spectrum (8–10). It is now well established that adaptation to changes in the dynamic TME induce acquisition of phenotypic plasticity (5, 66, 73, 75, 93, 94), a recently recognized cancer hallmark (13). With the advent of heterotypic spheroid models generated from various cell types, the dynamics of EMP can be studied and remaining questions can be addressed through longitudinal sampling and high-dimensional analysis of the tissue sections. The multicellular spheroids generated from aggregates of cancer cells and fibroblasts used in this study can be applied to study how fibroblasts affect the epithelial to mesenchymal plasticity of cancer cells, and to explore the best way to target the therapy-resistant clones in the context of EMP and additional signaling cues from the co-culture model to resist hypoxia and drug exposure. These physiologically relevant features represent in a superior manner the challenges facing successful targeting of the therapy-resistant clones *in vivo.* Approaches to target the most therapy-resistant populations could be performed by pre-treatment prior to co-culture or in the co-culture system. As various 3D models including spheroids and organoids are gaining popularity in pre-clinical studies, the major advantage of this heterotypic model is the ability to generate multicellular spheroids using cells of known genotype and phenotype, and with pre-determined ratios of cancer and stromal cells. The production is efficient and easily scalable, and the method is suitable for various downstream molecular readouts. Thus, the model holds significant potential in drug screening or drug penetration experiments and toxicity testing. By leveraging imaging mass cytometry or other multiplex imaging modalities, the cellular composition, spatial distribution, and protein expression in the spheroids can be studied without the need to dissociate to obtain single-cell data, providing mechanistic insights into the effects of treatments within a heterogenous, *in vitro* generated TME. Improved pre-clinical models for drug testing, along with incentives to openly reveal which of these models the pre-clinical data derive from, could provide a better foundation to select drug candidates for clinical testing, and thus ultimately improve pre-clinical to clinical translation (93).

## Data Availability Statement

The original contributions presented in the study are included in the article/**Supplementary Material**. Further inquiries can be directed to the corresponding author.

## Author Contributions

ML, GR, AR, SD, CE, NL, and AE performed most of the experiments. ML and AE analyzed data, made the figures, and participated in experimental design. AR and ML performed and analyzed the IMC experiments. KF and HD provided the ER cell culture system. GR, LS, JT, LA, JL, and AE secured funding, supported the work, and critically reviewed the experimental design. ML, GR, AR, JT, JL and AE wrote the manuscript. All authors critically reviewed and accepted the final version of the manuscript.

## Funding

The Research Council of Norway partly supported this work through its Centres of Excellence funding scheme, project number 223250 (CCBIO affiliates). ML was supported by a Ph.D. fellowship grant from Helse Vest RHF (the Western Norway Regional Health Authority). AR was supported by the Norwegian Research Council industrial PhD scheme PhD fellowship grant number 311397. JL was supported by grants from the Norwegian Research Council, project number: 301263, Norwegian Cancer Society (grant agreement No. KF190330). AE was supported by the FRIPRO Mobility Grant Fellowship from the Research Council of Norway, co-funded by the EU’s 7th Framework Programme’s Marie Curie Actions (MCA COFUND grant agreement No. 608695), and by grants from Legat for Forskning av Kreftsykdommer ved UIB and the Familien Blix fund.

## Conflict of Interest

Author AR was employed by BerGenBio.

The remaining authors declare that the research was conducted in the absence of any commercial or financial relationships that could be construed as a potential conflict of interest.

## Publisher’s Note

All claims expressed in this article are solely those of the authors and do not necessarily represent those of their affiliated organizations, or those of the publisher, the editors and the reviewers. Any product that may be evaluated in this article, or claim that may be made by its manufacturer, is not guaranteed or endorsed by the publisher.
