# Supplementary material for: Intrinsic Differences in Spatiotemporal Organization and Stromal Cell Interactions Between Isogenic Lung Cancer Cells of Epithelial and Mesenchymal Phenotypes Revealed by High-Dimensional Single-Cell Analysis of Heterotypic 3D Spheroid Models
Source: Front Oncol. 2022 Apr 22;12:818437. doi: 10.3389/fonc.2022.818437 (PMC9076321; doi:10.3389/fonc.2022.818437)
Supplement: Supplementary file 1 [file DataSheet_1.pdf]

Supplementary Material

Intrinsic differences in spatiotemporal organization and stromal cell interactions between isogenic lung cancer cells of epithelial and mesenchymal phenotypes revealed by high-dimensional single-cell analysis of heterotypic 3D spheroid models

Maria L. Lotsberg[1,2,3], Gro V. Røsland[1,2], Austin J. Rayford[1,2,4], Sissel E. Dyrstad[2], Camilla T. Ekanger[2], Ning Lu[1,2], Kirstine Frantz[5], Linda E. B. Stuhr[2], Henrik J. Ditzel[5,6], Jean Paul Thiery[1,7,8], Lars A. Akslen[1,3,9], James B. Lorens[1,2] and Agnete S. T. Engelsen[1,2*]

[1] Centre for Cancer Biomarkers (CCBIO), Department of Clinical Medicine, Faculty of Medicine, University of Bergen, Bergen, Norway
[2] Department of Biomedicine, Faculty of Medicine, University of Bergen, Bergen, Norway
[3] Department of Pathology, Haukeland University Hospital, Bergen, Norway
[4] BerGenBio, Bergen, Norway
[5] Institute of Molecular Medicine, University of Southern Denmark, Odense, Denmark
[6] Department of Oncology, Odense University Hospital, Odense, Denmark
[7] Guangzhou Laboratory, Guangzhou, China
[8] Gustave Roussy Cancer Campus, UMR 1186, Inserm, Universite´ Paris-Saclay, Villejuif, France
[9] Department of Clinical Medicine, Section for Pathology, Faculty of Medicine, University of Bergen, Bergen, Norway

* Correspondence: Dr. Agnete S. T. Engelsen E-mail: agnete.engelsen@uib.no

1 Supplementary Figures

Figure S1

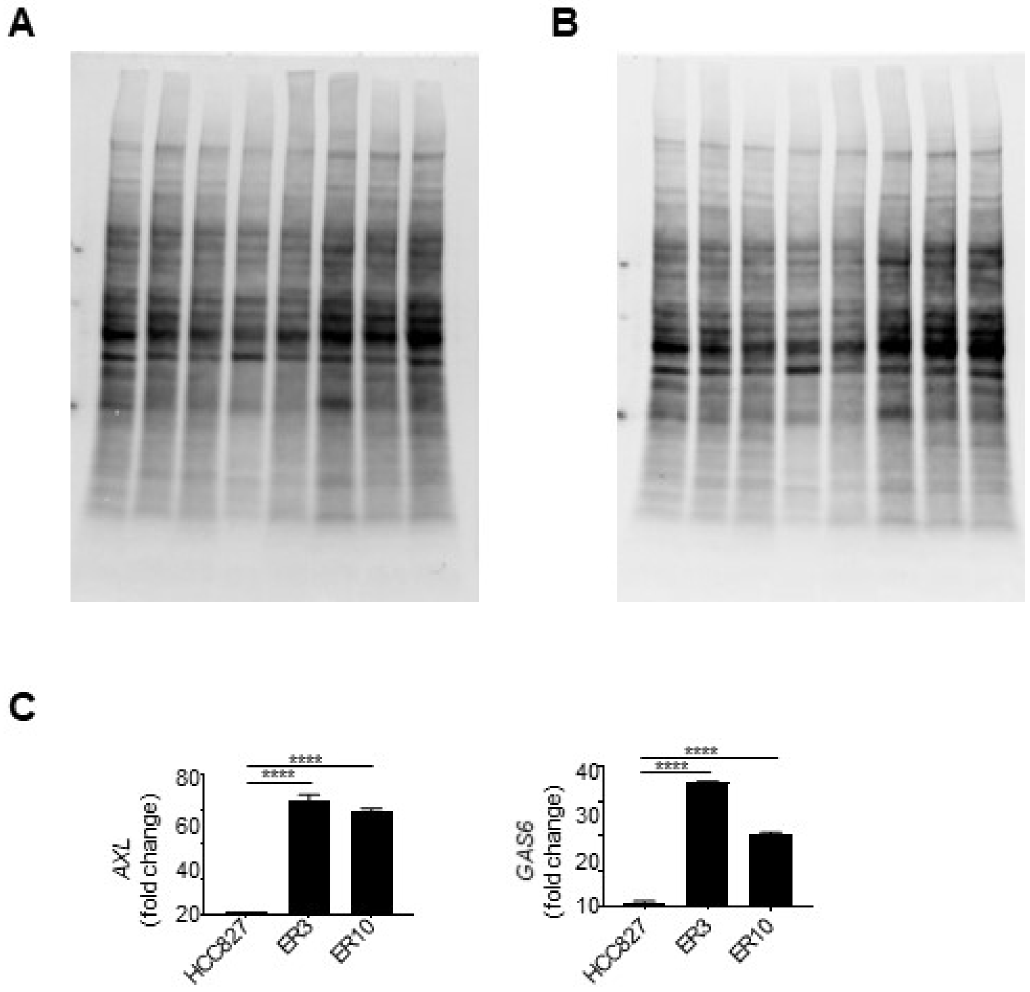

Figure S1: Total protein images for Western Blot quantification

Total protein images used for quantification of Western Blot in Figure 1C, quantified in Figure 1D and 1F. Total protein for CDH1 (E-cadherin) and VIM (vimentin) blot is shown in **(A)** and CDH2 (N-cadherin) **(B)**. **(C)** Expression of transcripts encoding *AXL,* and *GAS6,* assessed by RT-qPCR on cDNA prepared from HCC827 parental, ER3, and ER10 cells. RT-qPCR analyses were repeated n = 3 times, and representative results from one experiment with n = 3 technical replicates are presented in the figure as mean fold change +/- SD calculated by the $2^{-\Delta\Delta Ct}$ method. Two-way ANOVA followed by Tukey's multiple comparison test comparing ER3 and ER10 against the parental cell line was used to calculate statistical significance.

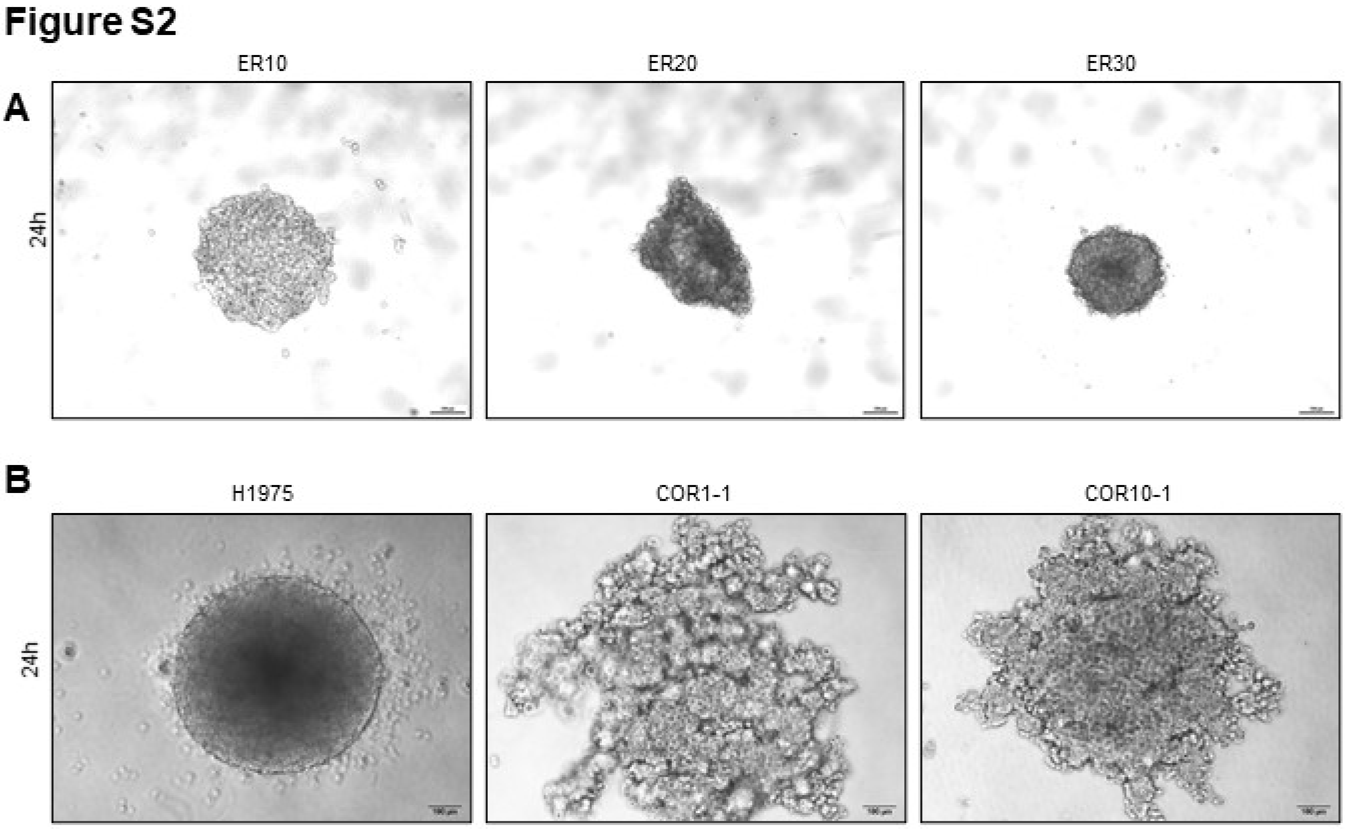

Figure S2: Spheroid formation in HCC827, COR1-1, COR10-1, ER10, ER20, and ER30.

(A) Nikon TE2000 images of ER10, ER20, and ER30 monoculture spheroids. Images were taken after 24 h using 4x objective. **(B)** Nikon TE2000 images of H1975, COR1-1, and COR10-1 monoculture spheroids. Images were taken after 24 h. 10x objective.

Figure S3

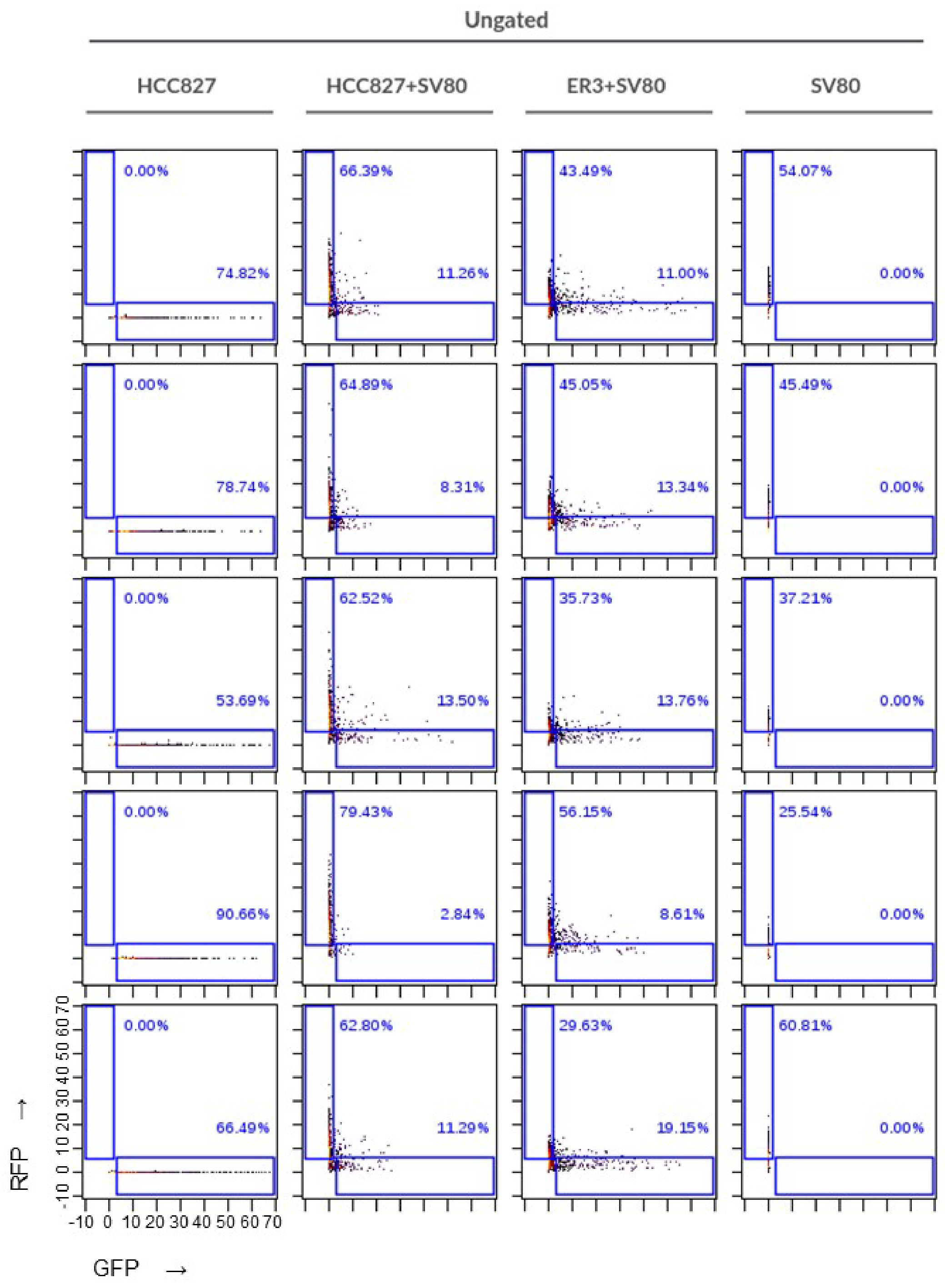

Figure S3: GFP+ and RFP+ gating strategy for untreated spheroids

Gating of GFP+ and RFP+ single-positive populations from the single-cell IMC data of untreated samples before visualizing channel intensities in the heatmap in Figure 7 Figure S4.

Figure S4

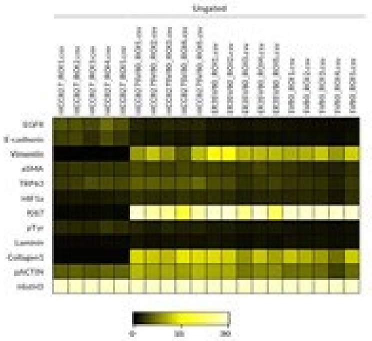

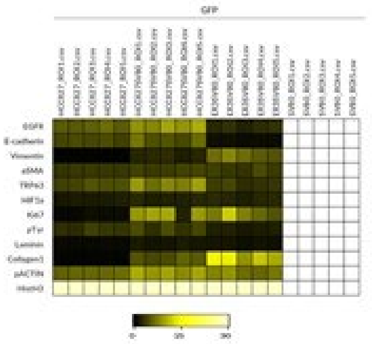

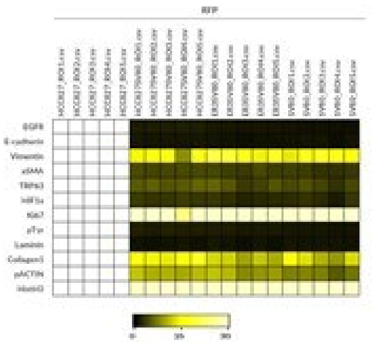

Statistics: Median of channel (varying by rows)

Ungated

	HCC827_ROI1.csv	HCC827_ROI2.csv	HCC827_ROI3.csv	HCC827_ROI4.csv	HCC827_ROI5.csv	HCC827SV80_ROI1.csv	HCC827SV80_ROI2.csv	HCC827SV80_ROI3.csv	HCC827SV80_ROI4.csv	HCC827SV80_ROI5.csv	ER35V80_ROI1.csv	ER35V80_ROI2.csv	ER35V80_ROI3.csv	ER35V80_ROI4.csv	ER35V80_ROI5.csv	SV80_ROI1.csv	SV80_ROI2.csv	SV80_ROI3.csv	SV80_ROI4.csv	SV80_ROI5.csv
EGFR	2.492	2.434	2.1	3.202	2.373	1.129	0.974	1.241	1.074	1.171	0.756	0.716	0.542	0.692	0.648	0.515	0.432	0.394	0.314	0.415
E-cadherin	1.839	1.998	1.285	2.204	1.611	0.871	0.658	0.804	0.644	0.815	0.492	0.478	0.351	0.433	0.393	0.42	0.442	0.453	0.302	0.506
Vimentin	0.0915	0.0869	0.0739	0.101	0.0849	4.489	5.795	4.572	2.699	5.589	7.385	7.494	5.539	5.459	5.473	5.095	5.21	5.874	4.502	6.087
aSMA	1.417	1.582	1.542	1.603	1.572	2.135	1.889	1.89	2.051	1.927	1.871	1.714	1.493	1.996	1.621	1.527	1.277	1.132	0.988	1.403
TRP63	1.678	1.767	2.257	2.192	2.067	2.786	2.739	2.551	2.639	3.015	2.223	2.154	1.435	1.733	1.925	1.589	1.537	1.448	0.933	1.696
HIF1a	0.641	0.741	0.655	0.786	0.884	1.525	1.3	1.284	1.542	1.389	1.434	1.226	0.89	1.138	1.185	1.12	1.214	1.08	0.649	1.405
Ki67	0.176	0.222	0.252	0.332	0.27	15.886	13.631	12.708	9.192	13.542	17.916	17.979	12.452	16.214	11.017	19.926	17.857	20.07	16.692	20.055
pTyr	1.249	1.158	1.089	1.554	1.259	1.095	0.833	1.125	0.941	1.025	0.779	0.821	0.61	0.719	0.708	0.546	0.503	0.487	0.376	0.493
Laminin	0.26	0.24	0.227	0.304	0.253	0.577	0.454	0.543	0.585	0.559	0.605	0.518	0.349	0.542	0.375	0.557	0.492	0.531	0.413	0.505
Collagen1	0.109	0.119	0.108	0.13	0.137	6.282	5.868	5.476	6.765	6.473	6.437	6.093	4.423	5.813	4.755	5.767	5.134	4.546	3.293	5.956
pACTIN	2.706	2.677	2.578	3.019	2.953	5.373	4.781	4.948	4.711	5.275	4.917	4.961	3.537	4.07	4.127	3.305	3.346	3.572	1.918	3.138
HistH3	45.568	44.781	43.671	58.062	47.443	105.136	69.432	97.83	156.234	73.787	41.985	43.853	32.403	56.216	32.701	45.525	36.598	25.909	18.306	48.61

GFP

	HCC827_ROI1.csv	HCC827_ROI2.csv	HCC827_ROI3.csv	HCC827_ROI4.csv	HCC827_ROI5.csv	HCC827SV80_ROI1.csv	HCC827SV80_ROI2.csv	HCC827SV80_ROI3.csv	HCC827SV80_ROI4.csv	HCC827SV80_ROI5.csv	ER35V80_ROI1.csv	ER35V80_ROI2.csv	ER35V80_ROI3.csv	ER35V80_ROI4.csv	ER35V80_ROI5.csv	SV80_ROI1.csv	SV80_ROI2.csv	SV80_ROI3.csv	SV80_ROI4.csv	SV80_ROI5.csv
EGFR	2.685	2.605	2.855	3.237	2.71	4.327	3.965	4.88	4.422	5.156	1.167	1.461	1.051	1.325	1.467					
E-cadherin	2.003	2.18	2.067	2.225	1.888	3.428	2.311	2.405	2.131	3.156	0.465	0.46	0.347	0.375	0.342					
Vimentin	0.0941	0.0894	0.0874	0.101	0.0862	1.084	1.196	1.289	0.81	1.117	3.194	3.925	3.18	2.684	2.423					
aSMA	1.497	1.614	1.642	1.614	1.611	1.916	1.711	1.768	1.95	1.995	1.858	1.608	1.493	2.141	1.756					
TRP63	1.875	1.958	2.407	2.259	2.335	4.526	3.202	3.937	3.694	4.685	1.798	2.02	1.329	1.472	1.591					
HIF1a	0.675	0.792	0.771	0.79	0.897	1.194	0.896	0.982	0.96	1.187	1.257	1.115	0.879	1.161	1.217					
Ki67	0.198	0.224	0.446	0.338	0.313	4.415	4.812	4.885	0.99	4.542	3.552	6.003	3.746	3.02	2.458					
pTyr	1.339	1.27	1.846	1.586	1.522	2.233	1.352	2.225	1.446	2.038	0.935	0.983	0.716	0.835	0.894					
Laminin	0.267	0.254	0.26	0.307	0.255	0.748	0.571	0.594	0.801	0.636	0.608	0.561	0.419	0.518	0.445					
Collagen1	0.111	0.127	0.124	0.13	0.134	2.428	2.074	2.207	2.885	2.648	7.195	6.982	4.305	5.767	5.052					
pACTIN	2.822	3.002	3.04	3.041	3.166	5.07	4.391	4.816	4.046	4.855	3.851	4.298	3.163	3.02	3.5					
HistH3	44.889	43.245	41.332	58.341	44.688	98.566	75.988	96.414	137.435	78.474	46.417	43.175	29.977	50.811	35.847					

RFP

	HCC827_ROI1.csv	HCC827_ROI2.csv	HCC827_ROI3.csv	HCC827_ROI4.csv	HCC827_ROI5.csv	HCC827SV80_ROI1.csv	HCC827SV80_ROI2.csv	HCC827SV80_ROI3.csv	HCC827SV80_ROI4.csv	HCC827SV80_ROI5.csv	ER35V80_ROI1.csv	ER35V80_ROI2.csv	ER35V80_ROI3.csv	ER35V80_ROI4.csv	ER35V80_ROI5.csv	SV80_ROI1.csv	SV80_ROI2.csv	SV80_ROI3.csv	SV80_ROI4.csv	SV80_ROI5.csv
EGFR						0.834	0.839	0.855	0.925	0.871	0.751	0.68	0.578	0.653	0.631	0.568	0.502	0.488	0.465	0.473
E-cadherin						0.717	0.578	0.66	0.651	0.671	0.593	0.539	0.406	0.467	0.458	0.525	0.598	0.591	0.406	0.588
Vimentin						7.29	7.743	7.853	4.17	8.129	9.956	9.374	8.156	7.1	8.453	6.855	8.24	8.615	7.894	7.708
aSMA						2.184	2.034	2.046	2.544	2.505	2.085	1.834	1.708	2.113	1.782	1.842	1.765	1.509	1.433	1.641
TRP63						2.631	2.79	2.469	2.628	2.909	2.956	2.499	1.717	1.982	2.446	2.146	2.359	1.999	1.479	2.079
HIF1a						1.739	1.518	1.589	1.781	1.596	1.611	1.357	1.026	1.207	1.232	1.301	1.502	1.267	0.804	1.582
Ki67						22.752	20.127	20.265	12.007	23.778	27.371	23.916	17.944	20.952	15.778	28.406	34.293	33.553	29.915	29.653
pTyr						0.949	0.79	0.966	0.895	0.884	0.859	0.866	0.689	0.727	0.742	0.677	0.641	0.651	0.57	0.574
Laminin						0.528	0.438	0.531	0.58	0.493	0.685	0.55	0.401	0.574	0.413	0.603	0.562	0.584	0.561	0.525
Collagen1						8.004	7.272	6.913	7.752	8.813	6.286	6.107	4.77	5.811	5.065	7.538	6.607	5.201	4.451	6.444
pACTIN						5.747	5.119	5.182	4.944	5.452	5.676	5.349	3.986	4.388	4.569	3.723	3.859	3.711	2.613	3.408
HistH3						110.72	73.805	105.761	125.989	78.173	45.428	47.004	38.29	60.255	36.334	64.11	51.098	36.029	31.643	60.892

Figure S4: Heatmaps of channel expression in untreated spheroids

Median fluorescence intensity values and heatmap visualization for the ungated, GFP+ and RFP+ populations of the untreated samples.

Figure S5

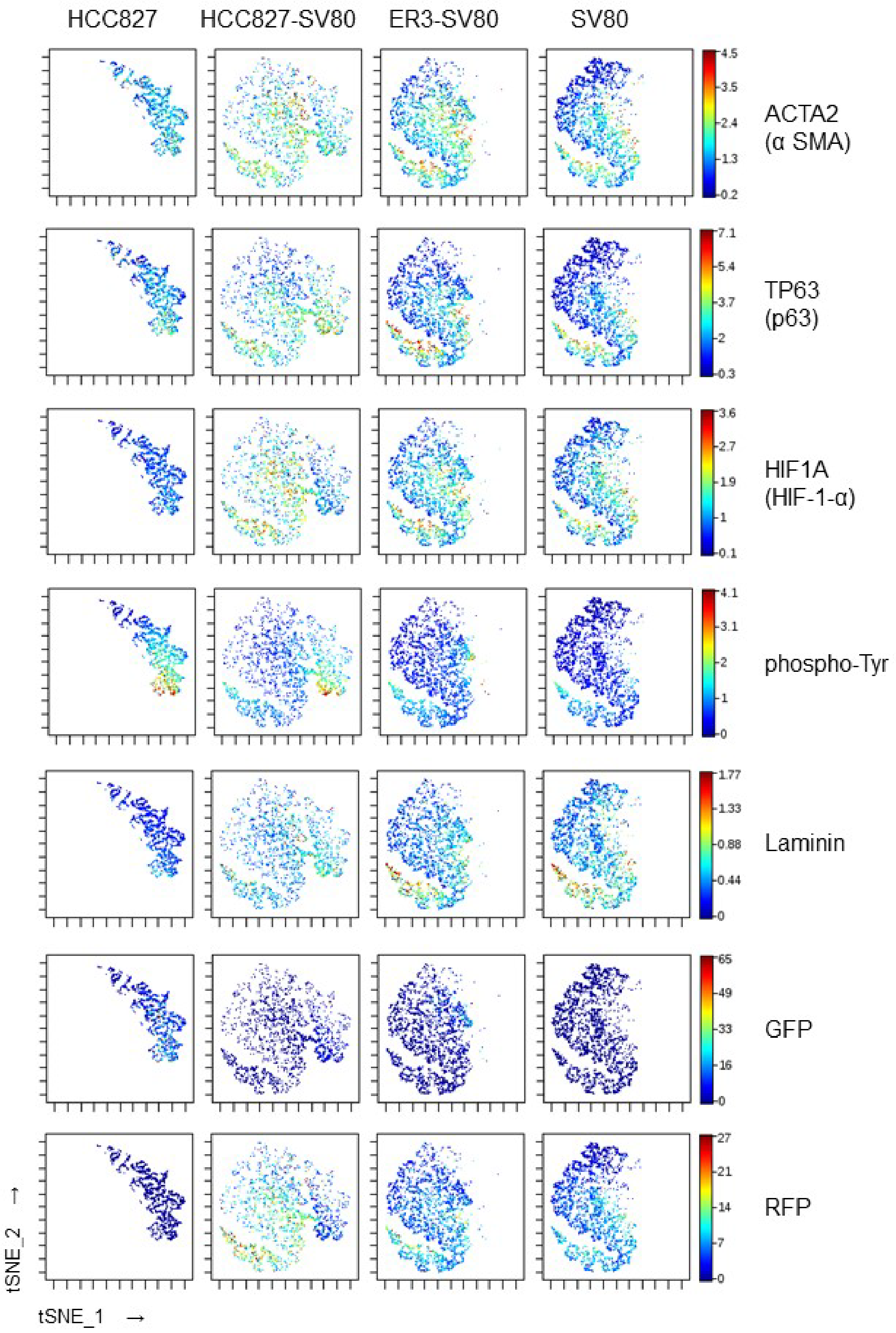

Figure S5: viSNE marker expression in untreated spheroids

Marker expression of ACTA2 (αSMA),TP63 (p63), HIF1A (HIF1α), phosphor-Tyr, Laminin, GFP, and RFP displayed on the viSNE plots from untreated samples.

Figure S6

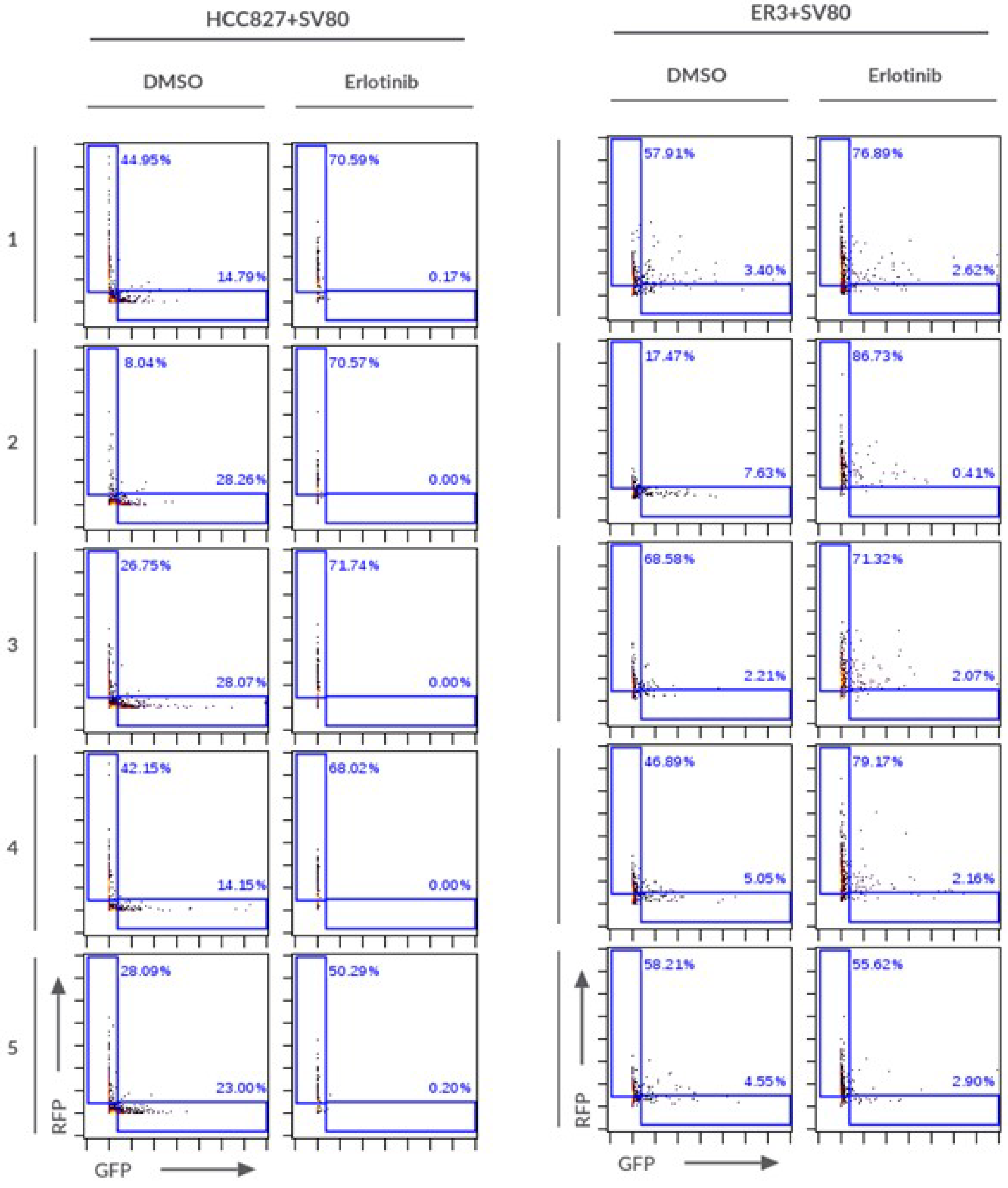

Figure S6: GFP+ and RFP+ gating strategy for treated spheroids

Gating of GFP+ and RFP+ single-positive populations from the single-cell IMC data from the DMSO and vehicle treated samples.

Figure S7

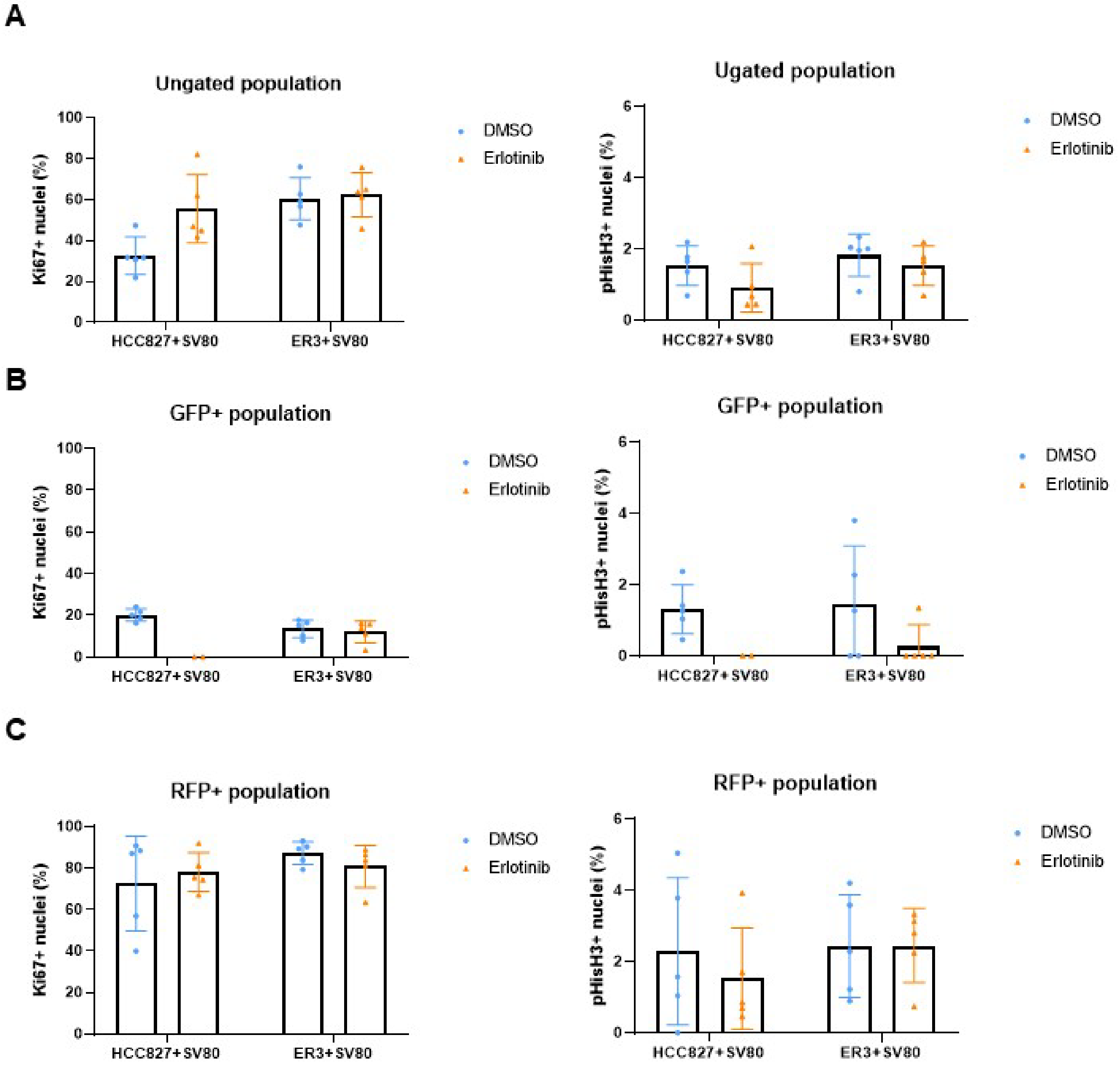

Figure S7: Percentage of Ki67 (MKI67) and phospho Histone H3 positive nuclei in vehicle (DMSO) and EGFRi (erlotinib) treated spheroids

Percentage of MKI67 (Ki67) positive (left) and phosphor-Histone H3 (pHisH3) positive (right) nuclei in the ungated **(A)**, GFP+ **(B)** and RFP+ **(C)** populations of vehicle (DMSO) and EGFRi (erlotinib) treated soheroids. No statistical significance was found by the Mann-Whitney U-test using cytobank to caluclate the statistics.

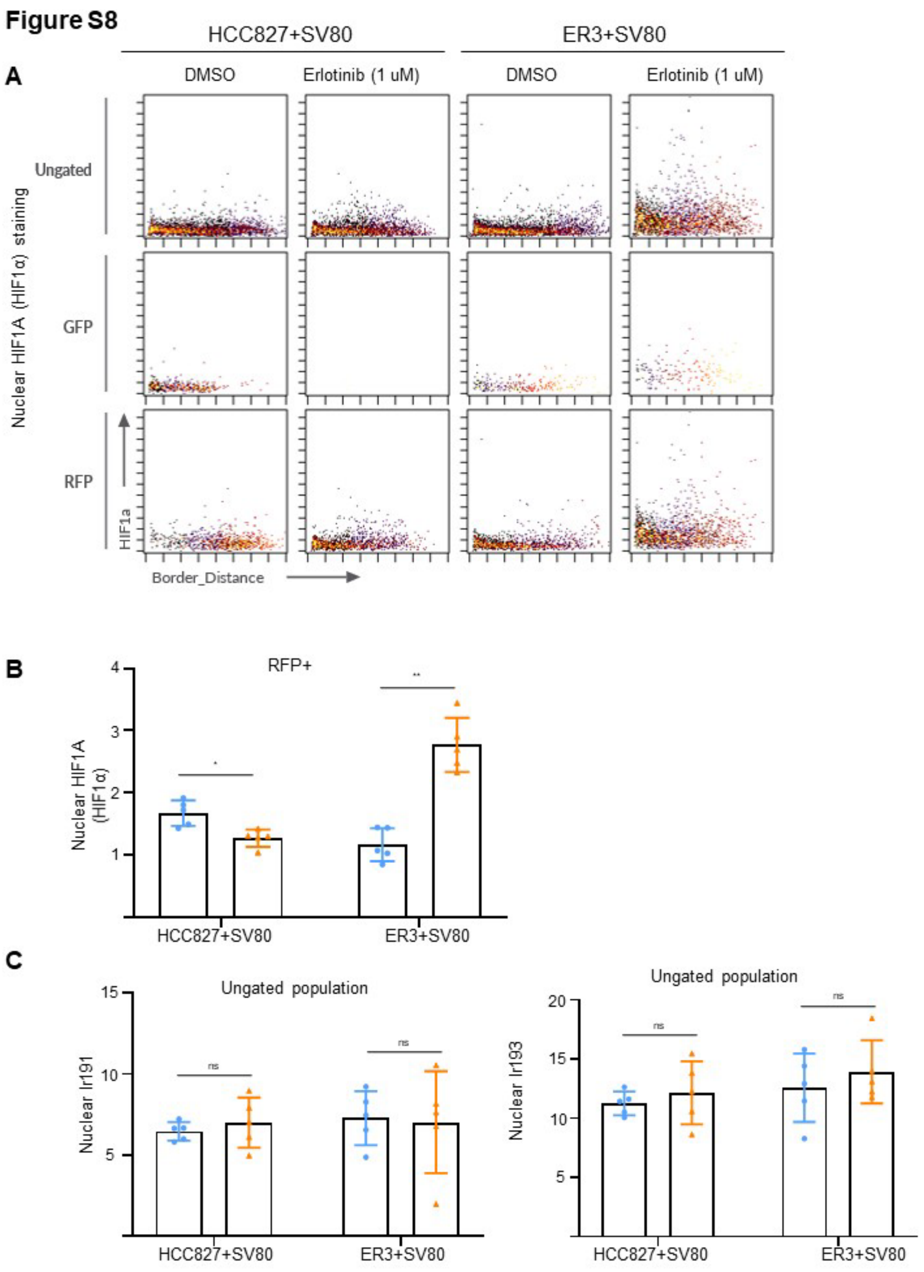

Figure S8: Scatter blots and channel expression of selected markers

(A) Scatter plots displaying nuclear expression (mean channel intensity within the nuclei mask) of HIF1A (HIF1α) plotted against distance to border in the ungated, GFP+ and RFP+ populations.

(B) Bar chart displaying quantified nuclear HIF1A (HIF1α) expression in the RFP+ population. Statistical significance in channel expression were calculated in cytobank using the Mann-Whithey U-test.

(C) Bar charts displaying the nuclear Ir191 (left) and Ir193 (right) expression in the ungated populations of erlotinib and DMSO treated samples. No statistical significance was found by the Mann-Whithey U-test using cytobank to caluclate the statistics.

Figure S9

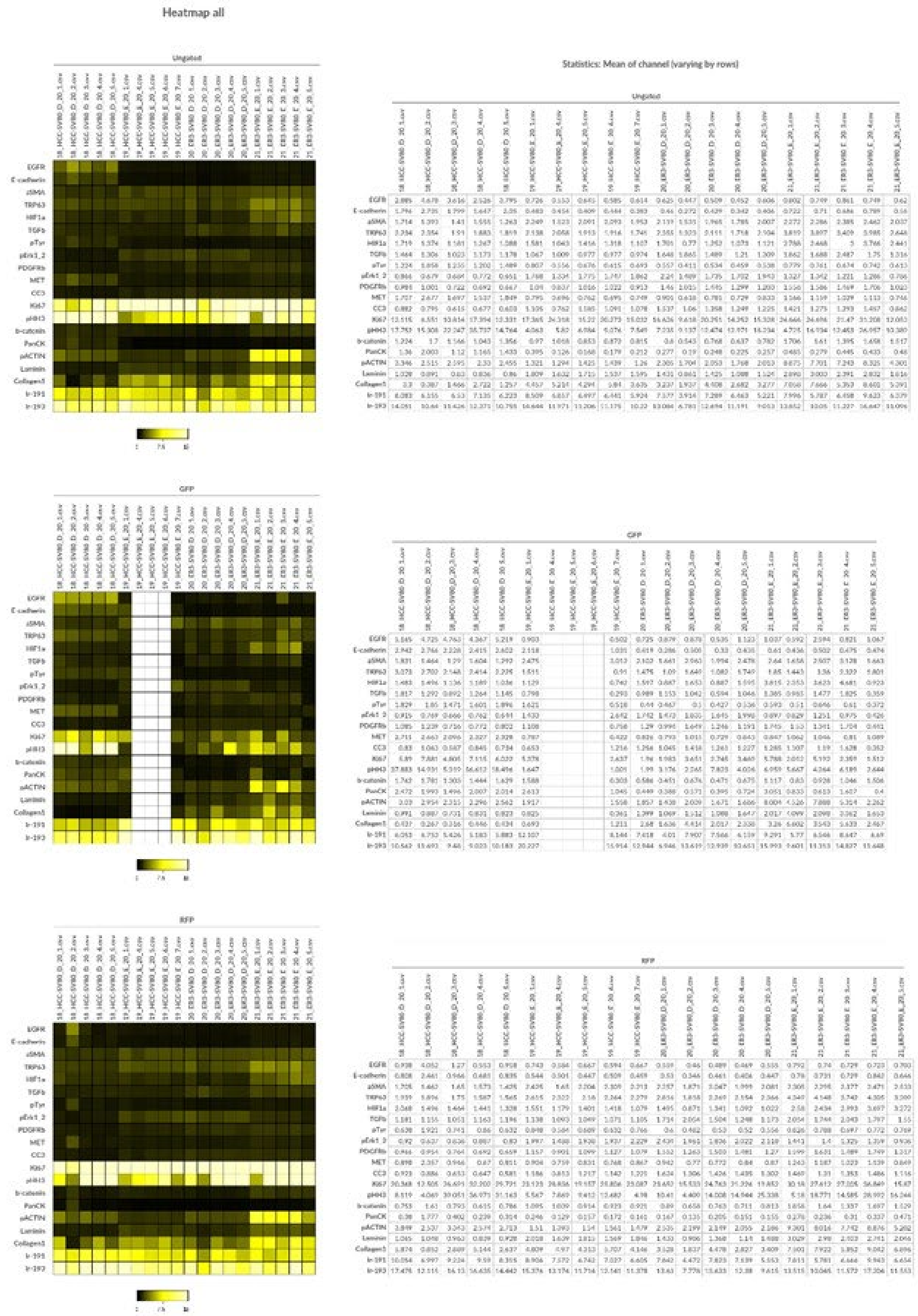

Figure S9: Heatmaps of marker expression of treated samples

Median fluorescence intensity values and heatmap visualization for the ungated, GFP+ and RFP+ populations of the vehicle (DMSO, D) and erlotinib (E) treated samples.

Figure S10

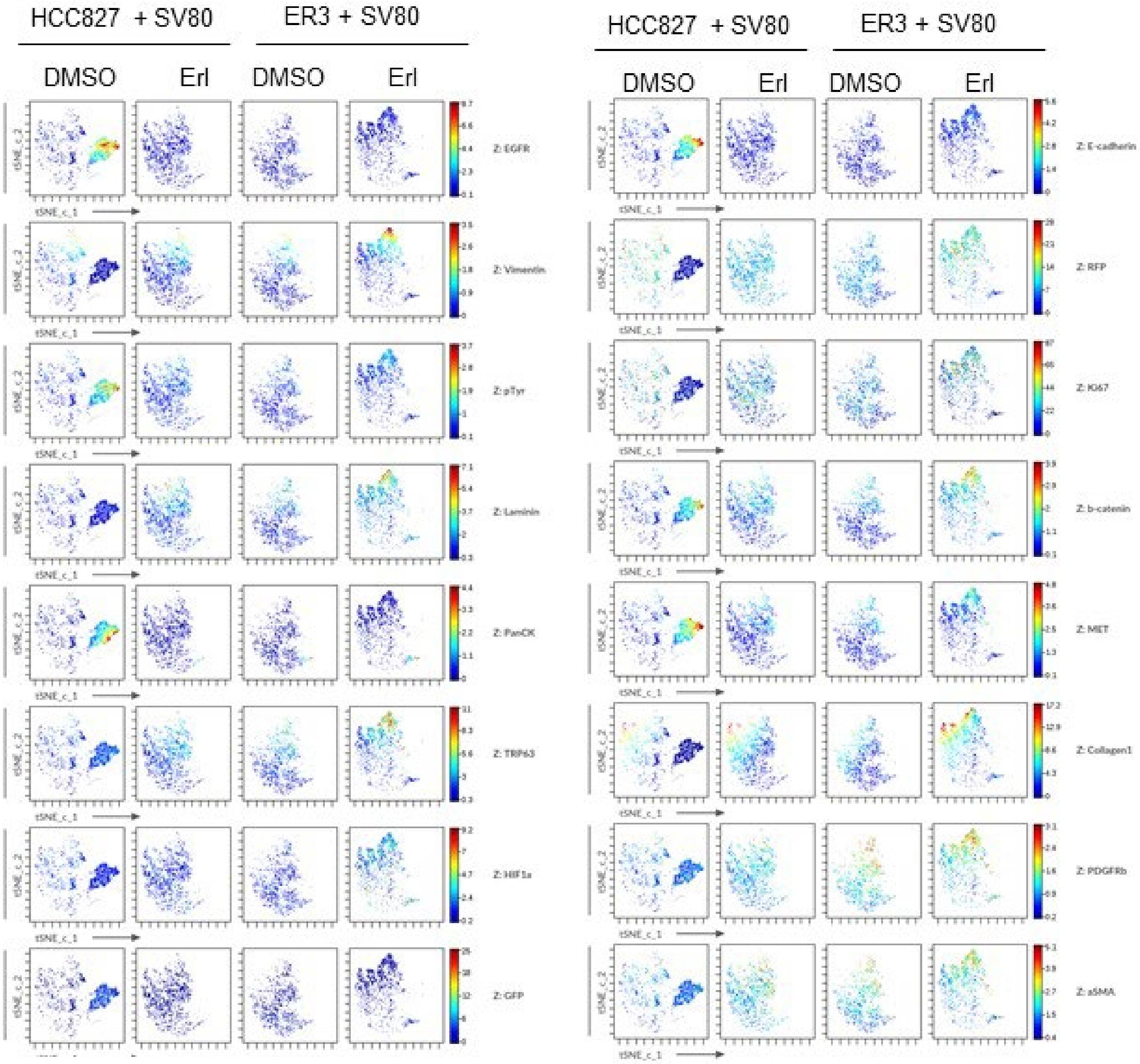

Figure S10: ViSNE marker expression of treated samples

Marker expression displayed on the viSNE plots for erlotinib and vehicle control (DMSO) treated samples.

2 Supplementary Videos

Video S1:

IncuCyte formation assay of ER3-GFP spheroids.

Video S2:

IncuCyte formation assay of SV80-dsRed spheroids.

Video S3:

IncuCyte formation assay of ER3-GFP + SV80-dsRed heterotypic co-culture spheroids.

Video S4:

3D reconstruction in IMARIS of ER3-GFP + SV80-dsRed heterotypic co-culture spheroids imaged by Zeiss confocal microscopy and counterstained with Hoechst nuclear dye.
